# Enrichment of hydroxylated C24- and C26-acyl-chain sphingolipids mediates PIN2 apical sorting at *trans*-Golgi network subdomains

**DOI:** 10.1038/ncomms12788

**Published:** 2016-09-29

**Authors:** Valérie Wattelet-Boyer, Lysiane Brocard, Kristoffer Jonsson, Nicolas Esnay, Jérôme Joubès, Frédéric Domergue, Sébastien Mongrand, Natasha Raikhel, Rishikesh P. Bhalerao, Patrick Moreau, Yohann Boutté

**Affiliations:** 1 UMR 5200 Membrane Biogenesis Laboratory, CNRS-University of Bordeaux, Bâtiment A3 - INRA Bordeaux Aquitaine, 71 Avenue Edouard Bourlaux - CS 20032, 33140 Villenave d'Ornon, France; 2 Bordeaux Imaging Center, UMS 3420 CNRS, US4 INSERM, University of Bordeaux, 33000 Bordeaux, France; 3 Umeå Plant Science Centre, Department of Forest Genetics and Plant Physiology, Swedish University for Agricultural Sciences, SE-901 83 Umeå, Sweden; 4 Center for Plant Cell Biology, Department of Botany and Plant Sciences, University of California, Riverside, California 92521, USA; 5 College of Science, KSU, 11451 Riyadh, Saudi Arabia

## Abstract

The post-Golgi compartment *trans*-Golgi Network (TGN) is a central hub divided into multiple subdomains hosting distinct trafficking pathways, including polar delivery to apical membrane. Lipids such as sphingolipids and sterols have been implicated in polar trafficking from the TGN but the underlying mechanisms linking lipid composition to functional polar sorting at TGN subdomains remain unknown. Here we demonstrate that sphingolipids with α-hydroxylated acyl-chains of at least 24 carbon atoms are enriched in secretory vesicle subdomains of the TGN and are critical for *de novo* polar secretory sorting of the auxin carrier PIN2 to apical membrane of *Arabidopsis* root epithelial cells. We show that sphingolipid acyl-chain length influences the morphology and interconnections of TGN-associated secretory vesicles. Our results uncover that the sphingolipids acyl-chain length links lipid composition of TGN subdomains with polar secretory trafficking of PIN2 to apical membrane of polarized epithelial cells.

## Abstract (web summary)

Sphingolipids in the *trans*-Golgi network have been implicated in polar trafficking. Here Wattelet-Boyer *et al*. show that hydroxylated C24- and C26-acyl-chain sphingolipids are enriched in *trans*-Golgi network subdomains that are critical for polar sorting of the PIN2 auxin carrier in plant cells.

## Graphical abstract

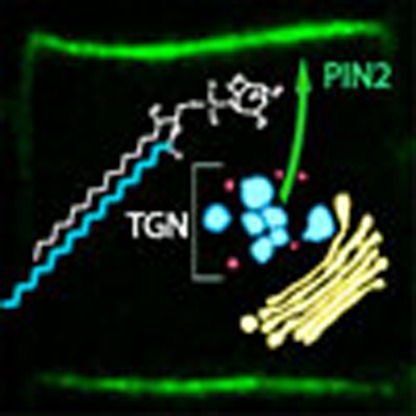

The *trans*-Golgi network (TGN) is a heterogeneous tubulo-vesiculated post-Golgi structure, which matures from the Golgi, with distinct membrane subdomains that aid in the sorting and segregation of cargos to different cell compartments such as the plasma membrane (PM), vacuole and endosomes. Importantly, TGN plays a central role in trafficking of cargo that is deposited to highly specialized domains of the cell, for example, lipids and proteins destined to apical membrane through polar delivery^1^,^2^,^3^. Examples of such polar transport to apical membrane include the influenza virus haemagglutinin of Madin–Darby canine kidney cells and PIN2, an auxin efflux carrier localized to the apical membrane of root epithelial cells in plants^4^,^5^,^6^. Sphingolipids (SLs) are proposed to play a key role in polar delivery of proteins to PM. This suggestion is based on several lines of evidence. For example, during the course of polarization of animal epithelial cells, fatty acids (FAs) chain length and hydroxylation of SLs increase^7^. In *Caenorhabditis elegans*, apico-basal polarity conversion is observed in mutants for FAs and SLs biosynthetic enzymes^8^. Moreover, in budding yeast, TGN-derived vesicles involved in polar exocytosis are enriched in SLs and sterols^9^. Although these data support a role of SLs in polarity and polar delivery of proteins to PM, how these lipids mediate polar secretory sorting from the TGN is currently not well understood.

In this study, we used the polarized epithelium of roots from the plant model *Arabidopsis thaliana* and took advantage of the well-established auxin efflux carrier PIN2 protein, for which the localization is polar at apical membrane of root epithelial cells^6^. In plants, coordination of morphogenesis heavily relies on the phytohormone auxin. Polar auxin transport allows directionality of short distance auxin fluxes and mediates concentration of auxin at defined areas of plants to modulate growth patterns and axes^10^,^11^,^12^,^13^,^14^. Modulation of root growth axis in response to a change in gravity (gravitropism) is known to rely on apical polarity of PIN2 in root epidermal cells^6^,^15^,^16^. Hence, attenuation of root gravitropism is a good readout for possible defects in apical polarity in root epithelial cells. PIN2 polarity at apical membrane is known to hinge on PM recycling, defined endocytosis at the edge of the polar domain and clustering of PIN2 in small domains of PM^5^. PM recycling of PIN2 is partly mediated by the Exo70A1 and sec8 proteins of the exocyst complex and the nucleotide exchange factor for ARF GTPases (ARF-GEF) GNL1, which localizes to the Golgi apparatus^17^,^18^. Interestingly, PIN2 recycling at apical membrane is also dependent on the ARF GTPase ARF1A1C/BEX1, which localizes both to the Golgi apparatus and to TGN^19^. Hence, Golgi apparatus and TGN appear to be playing a central role in PM recycling of PIN2 and its apical polarity at PM. Intriguingly, fluorescence recovery after photobleaching experiments indicate that PIN2 is apically delivered through uncharacterized polar exocytosis/delivery mechanisms^5^.

Here we show that, in *Arabidopsis*, subdomains of TGN are distinguished by differences in SL and sterol composition. We show that TGN-associated secretory vesicles (SVs) are enriched in sterols and α-hydroxylated very-long-chain FAs (hVLCFAs) containing 24 (h24) or 26 (h26) atoms of carbon. Inversion of the FAs≥24/FAs≤24 ratio within the pool of SLs, without interfering with global quantity of SLs, results in a loss of PIN2 polarity at apical membrane and *de novo* secretory blockage of PIN2 in SVs. Moreover, this inversion also has impacts on the morphology of TGN-associated SVs and tubular membrane interconnections established between SVs. Altogether, our results reveal a role for the length of α-hydroxylated acyl chains of SLs, enriched at TGN, in secretory trafficking to apical membrane of polarized epidermal cells.

## Results

### SVs subdomain of TGN is enriched in hVLCFAs of SLs

**Figure 1:**
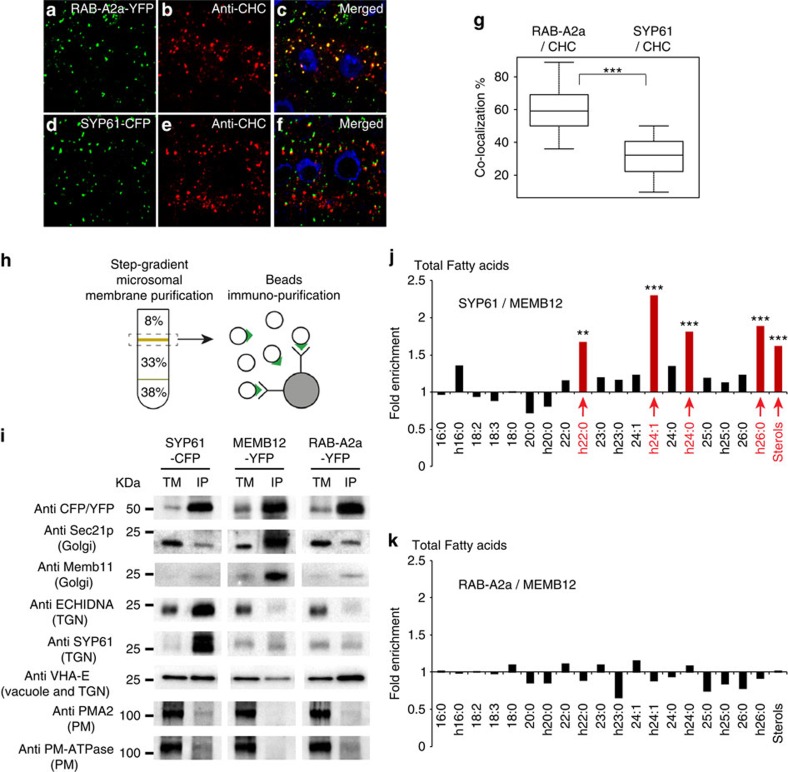
TGN subdomain labelled by SYP61 is enriched in hVLCFAs and sterols as compared with Golgi or TGN subdomain RAB-A2a enriched for clathrin. (**a**–**g**) Immunolocalization of CHC (**b**,**e**) in *Arabidopsis* root epithelial cells expressing either the TGN marker RAB-A2a–YFP (**a**) or the TGN marker SYP61–CFP (**d**). Strong co-localization between RAB-A2a and CHC is detected in merged images (**c**), whereas weaker co-localization is visualized between SYP61 and CHC (**f**,**g**). Statistical analysis show highly significant difference between RAB-A2a/CHC co-localization and SYP61/CHC co-localization (*n*=50 cells distributed over 10 roots for each experiment, 3 biological replicates). (**h**) Immunopurifications of SYP61–CFP-, MEMB12–YFP- and RAB-A2a–YFP-labelled compartments were performed by incubating a step-gradient-purified TM fraction with beads coated with anti-CFP/YFP antibodies. (**i**) Western blottings on IP SYP61–CFP-, RAB-A2a–YFP- and MEM12–YFP-labelled intact vesicles. IP, beads-IP fraction; TM, input, step-gradient-purified TM fraction. As compared with the input (TM), anti-CFP/YFP antibodies revealed that all protein markers (SYP61–CFP, MEMB12–YFP or RAB-A2a–YFP) are enriched in their targeted IP compartments. Sec21p and MEMB11 markers of the Golgi apparatus are enriched in IP MEMB12–YFP-labelled Golgi but not in IP SYP61–CFP-labelled TGN or RAB-A2a–YFP-labelled TGN. The ECHIDNA and SYP61 markers of TGN-associated secretory vesicles are enriched in IP SYP61–CFP-labelled TGN but not in MEMB12–YFP-labelled Golgi or RAB-A2a–YFP-labelled TGN. V-ATPase VHA-E, which traffic through the TGN, is not enriched in SYP61-immunopurified or MEMB12-immunopurified fraction but is slightly enriched in IP RAB-A2a–YFP-labelled TGN. The PMA2 and PM-ATPase markers for PM are not enriched in any IP compartments. (**j**,**k**) Acyl-chain composition of the total pool of FAs contained in IP fractions. (**j**) As compared with MEMB12–YFP-labelled Golgi, SYP61–CFP-labelled TGN shows a significant enrichment (about 2-fold, *n*=11 IPs for each compartment, 11 biological replicates for each compartment) in hVLCFAs h22:0, h24:1, h24:0, h26:0 and sterols. (**k**) As compared with MEMB12–YFP-labelled Golgi, RAB-A2a–YFP-labelled TGN does not display any enrichment in hVLCFAs or sterols (*n*=11 IPs for each compartment, 11 biological replicates for each compartment). Statistics were done by two-sided Wilcoxon's rank-sum test, **P-value<0.01, ***P-value<0.001. All scale bars, 5 μm.

To investigate the role of SLs in polar exocytosis, we analysed distribution of SLs in TGN. In *Arabidopsis* root cells, the TGN population labelled by the syntaxin SYP61 is distinct from another TGN population labelled by the RAB-GTPase RAB-A2a^20^,^21^,^22^,^23^. Ultra-structural analyses by electron tomography have shown that SYP61 localizes to SVs at TGN^24^. Interestingly, a conserved protein ECHIDNA (ECH) strongly co-localizes with SYP61 but weakly with clathrin heavy chain (CHC)^22^, whereas RAB-A2a, but not SYP61, strongly co-localized with CHC (Fig. 1a–g). Hence, TGN-associated SVs are marked by SYP61/ECH, whereas TGN-associated clathrin-coated vesicles (CCVs) are marked by RAB-A2a and represent two distinct subdomains of TGN. We used an immuno-isolation procedure yielding highly purified intact TGN compartments^25^ in transgenic *Arabidopsis* plants expressing either the TGN-localized syntaxin SYP61 fused to the fluorescent tag cyan fluorescent protein (CFP)^26^ or RAB-A2a fused to yellow fluorescent protein (YFP)^20^ (Fig. 1h). In addition, a Golgi marker, the Qb-SNARE Membrin12 (MEMB12) fused to YFP was used to isolate Golgi compartment^27^,^28^. We performed co-localization analyses between MEMB12–YFP and the Golgi marker MEMB11, for which we characterized the localization at the Golgi apparatus previously by electron microscopy^29^. Our results show a strong co-localization between MEMB12–YFP and MEMB11 (Supplementary Fig. 1a–c), indicating that MEMB12–YFP is a good marker to isolate Golgi apparatus. Moreover, co-localization between MEMB12–YFP and either ECH (Supplementary Fig. 1g–i) or CHC (Supplementary Fig. 1m–o) is low. Thus, Golgi labelled with MEMB12–YFP/MEMB11 are distinct from TGN-associated SVs labelled by SYP61–CFP/ECH and from TGN-associated CCVs labelled by RAB-A2a–YFP/CHC.

Western blottings of SYP61–CFP, MEMB12–YFP and RAB-A2a–YFP immunopurified (IP) fractions, loaded to equal amount (Supplementary Fig. 2a), using anti-green fluorescent protein (GFP) antibodies showed enrichment of targeted compartments in the IP output (beads coupled to GFP antibodies, IP) compared with the IP input (total membrane (TM) fraction; Fig. 1i). We estimated the fold enrichment by quantifying the mean intensity of signals obtained on western blotting and evaluated that SYP61–CFP compartment was 9.5±0.2 (± indicates s.d., *n*=3) fold enriched, MEMB12–YFP was 9.2±0.4 (± indicates s.d., *n*=3) fold enriched and RAB-A2a was 9.3±0.3 (± indicates s.d., *n*=3) fold enriched, as compared with the IP input (TM fraction). Importantly, TGN markers ECH (anti-ECH) and SYP61 (anti-SYP61) were enriched in SYP61–CFP IP fraction, whereas ECH was neither detected in MEMB12–YFP nor in RAB-A2a–YFP IP fractions (Fig. 1i) in agreement with previous data showing minimal co-localization between ECH and RAB-A2a or Golgi markers^21^. Furthermore, SYP61 protein was only weakly detected in MEMB12–YFP or RAB-A2a–YFP IP fractions (Fig. 1i), whereas the Golgi marker SEC21 (anti-SEC21) and the Golgi marker MEMB11 (anti-MEMB11) were both enriched in MEMB12–YFP IP fraction and only weakly detected in SYP61–CFP or RAB-A2a–YFP IP fractions (Fig. 1i). These results strongly suggest the successful separation of the TGN subdomain SYP61–CFP/ECH/SVs from either the TGN subdomain RAB-A2a–YFP/CCVs or the Golgi apparatus by the IP isolation protocol. We also tested the presence of V-ATPase of the VHA-E family using an antibody recognizing VHA-E1, VHA-E2 and VHA-E3 subunits, which localize both to vacuoles and TGN, reflecting the secretion pathway to vacuoles through TGN^30^. We detected signals in SYP61, RAB-A2a or MEMB12 IPs, but as compared with the IP input (TM fraction) we did not detect any enrichment of VHA-E in SYP61 IPs, whereas VHA-E was even strongly depleted in Golgi MEMB12 IPs (Fig. 1i). Interestingly, enrichment of VHA-E was detected in RAB-A2a IPs, which is consistent with the description of clathrin-dependent trafficking from TGN to vacuoles^31^,^32^ and our previous finding that RAB-A2a strongly co-localizes with clathrin (Fig. 1a–c,g). To check PM contamination we used two different ATPases of the PM: PMA2 and PM-H^+^-ATPase. As compared with IP input (purified TM fraction), very weak signal was detected for PMA2 and PM-H^+^-ATPase in SYP61, MEMB12 and RAB-A2a IP output fractions (beads coupled to GFP antibodies) (Fig. 1i). These results confirmed that PM contaminations were negligible in isolated SYP61, RAB-A2a or MEMB12 populations. Altogether, these results indicate that IP allows high level of separation of SYP61/SVs, RAB-A2a and MEMB12/Golgi compartments.

**Figure 2:**
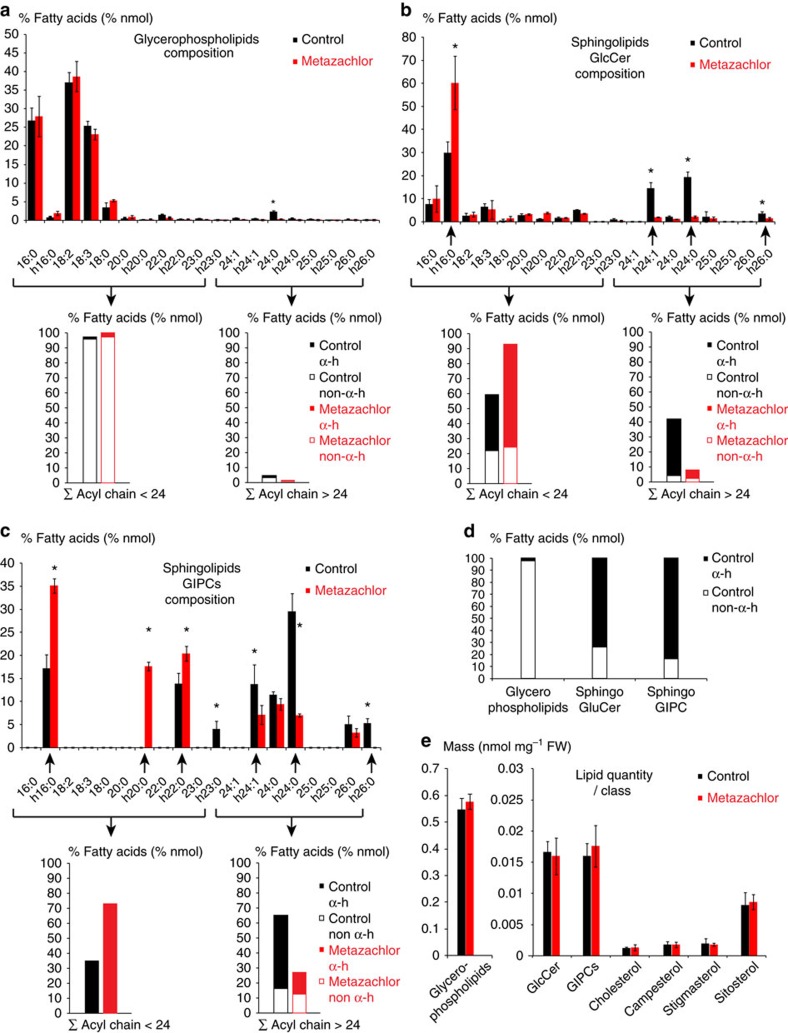
hVLCFAs are almost specific of SLs and 50 nM metazachlor alters the composition of VLCFAs in the pool of SLs. In untreated *Arabidopsis* roots, the pool of FAs of glycerophospholipids (**a**) contains very few hFAs (**d**). Metazachlor significantly reduces the 24:0-containing FAs but does not alter the global composition of FAs of glycerophospholipids, which are mainly composed of C16- and C18-containing FAs. Arrowed brackets display the sum of<24 carbon atom-containing FAs and>24 carbon atom-containing FAs. Each histogram is further divided in hFAs (α-h) and non-h FAs (non-α-h). The ratio between<24- and>24-FAs is not altered in the glycerophospholipids pool. (**b**,**c**) Contrastingly to glycerophospholipids, in untreated roots, FAs of GlcCer (**b**) and GIPCs (**c**) contain a substantial amount of hFAs (**d**). Interestingly, metazachlor strongly reduces α-hydroxylated h24:1, h24:0 and h26:0 and increases α-hydroxylated h16:0, h20:0 and h22:0 FAs of GlcCer and GIPCs pools. Arrowed brackets display the sum of<24 carbon atom-containing FAs and>24 carbon atom-containing FAs. Each histogram is further divided in hFA α-h and non-hFA non-α-h. The ratio between<24- and>24-FAs is drastically inverted in both GlcCer and GIPCs pool. (**e**) Metazachlor neither alters the global quantity of FAs of the glycerophospholipids pool nor the global quantity of FAs of the GlcCer and GIPCs pools. Moreover, global quantities of individual sterols are not affected by metazachlor. Statistics were done by two-sided Wilcoxon's rank- sum test, *P-value<0.05, *n*=4 for each experiment, 4 biological replicates. Errors bars are s.d.

Following the successful isolation of the SYP61-, RAB-A2a- and MEMB12-enriched IP fractions, we quantified lipids in these fractions by gas chromatography coupled to a mass spectrometer (GC-MS). As compared with GC coupled to a Flame ionization detector (GC-FID), we found that, when running the same mass (50 μg) of individual FAs with various chain length in either GC-MS or GC-FID, areas of corresponding FA peaks were highly similar between GC-MS and GC-FID for all FAs chain length tested (Supplementary Fig. 2b). This validates the use of GC-MS to quantify FAs with diverse chain length. Our results show strong enrichment of the hVLCFAs h22:0, h24:1, h24:0 and h26:0 in SYP61–CFP IP fraction as compared with Golgi/MEMB12–YFP IP fraction (Fig. 1j). Enrichment of sterols was also observed in SVs/SYP61–CFP IP fraction compared with Golgi/MEMB12–YFP IP fraction (Fig. 1j). Interestingly, sterol and FAs composition of RAB-A2a–YFP were not different from that of the Golgi fraction (Fig. 1k). To identify which lipids contain h22:0, h24:1, h24:0 and h26:0, we then analysed FA composition of various lipid pools in *Arabidopsis* root. The FAs of glycerophospholipids contained only traces (2.3%) of α-hydroxylated FAs (hFAs; Fig. 2a,d). In contrast to glycerophospholipids, FAs of the SL glucosylceramide (GlcCer) contained 74% of hFAs (Fig. 2b,d). As in plants, glycosyl-inositol-phosphoryl-ceramides (GIPCs) are the most preponderant SLs^33^, we also analysed FA composition within the pool of GIPCs. Our results indicated that in *Arabidopsis* root, FAs of GIPCs contained 83.5% of hFAs (Fig. 2c,d). In conclusion, our results suggest an enrichment of GIPCs and GluCer rather than glycerophospholipids in SYP61 compartment of the TGN and, importantly, the h22:0, h24:1, h24:0 and h26:0 appears to dominate in the GIPCs and GluCer in TGN/SYP61 compartment in the *Arabidopsis* roots.

### Metazachlor alters hVLCFAs in the pool of SLs

**Figure 3:**
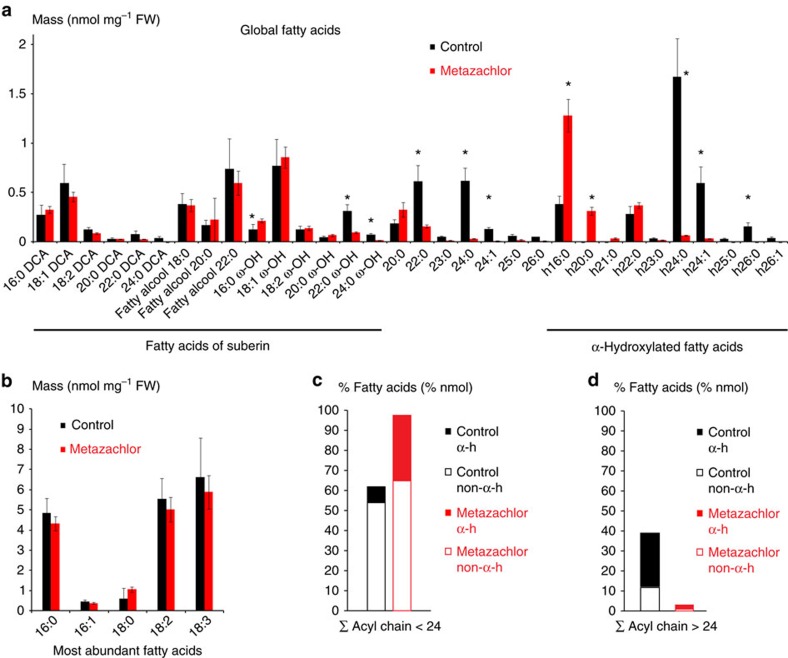
Global FAs analysis reveals that metazachlor reduces all types of >24-FAs. (**a**) Global FAs of *Arabidopsis* roots in untreated (black) and metazachlor-treated (red) conditions. Compounds of the root suberin, dicarboxylic acid DCA and fatty alcohols are not altered by a 50 nm metazachlor treatment. However, ω-hVLCFAs, which are components are the root suberin, are significantly reduced on metazachlor. Non-hydroxylated C22 and C24-FAs, which are more abundant in GIPCs than in glycerophospholipids, are significantly reduced on metazachlor. hVLCFAs h22:0, h24:0, h24:1, h25:0, h26:0 and h26:1, almost exclusively present in GIPCs, are strongly reduced on metazachlor. Accumulation of h16:0 and h20:0 is observed on metazachlor. (**b**) Contrastingly to VLCFAs, metazachlor does not alter the quantity of C16- and C18-containing FAs (*n*=4 for each experiment, 4 biological replicates). (**c**,**d**) Sums of <24 carbon atom-containing FAs (**c**) and>24 carbon atom-containing FAs (**d**). Each histogram is further divided in hFAs (α-h) and non-hFAs (non-α-h). On metazachlor, the ratio between <24- and >24-FAs is drastically inverted. Statistics were done by two-sided Wilcoxon's rank-sum test, *P-value<0.05, *n*=4 for each experiment, 4 biological replicates. Error bars are s.d.

Our data indicate that hVLCFAs are a characteristic defining feature of TGN/SYP61 compartments. Hence, we searched for pharmacological tools enabling us to modify chain-length composition of hFAs in SLs. Metazachlor, a chloracetamide-based herbicide, is a known inhibitor of VLCFA synthesis that directly targets the 3-ketoacyl CoA synthase (KCS) enzymes of the elongase complex, which condense two carbons at a time on a preexisting FAs chain^34^. In this study, we found that metazachlor was an ideal tool to alter VLCFAs without decreasing the total quantity contained in each pools of lipids. Global analyses of FAs composition in *Arabidopsis* roots of 5-day-old seedlings revealed that 50 nM metazachlor strongly decreases h24:0, h24:1, h26:0 and h26:1 hFAs, whereas we observed an accumulation of h16:0 and h20:0 hFAs (Fig. 3a). Metazachlor also decreases non-hydroxylated 22:0 and 24:0 FAs and ω-hydroxylated 22:0 and 24:0 FAs (22:0 ω-OH and 24:0 ω-OH) (Fig. 3a). No effects of 50 nM metazachlor treatment is observed on C16- and C18-containing FAs (Fig. 3b), which are the most abundant FAs, suggesting that the *de novo* FAs synthesis is not altered by metazachlor. Our results show that when we summed up all types of FAs <24 (Fig. 3c) and all type of FAs >24 (Fig. 3d) atoms of carbon, we could clearly see that the FAs <24/FAs >24 ratio is inverted on metazachlor (Fig. 3c,d). The ratio FAs <24/FAs >24 (Fig. 3c,d) also suggests that hFAs are the most altered by metazachlor. As we show in Fig. 2d that hFAs are almost exclusively present in SLs, we guessed that metazachlor would target more the pool of SL than other lipids. Hence, to check how specific metazachlor is towards distinct classes of lipids, we next analysed individual classes of lipids in *Arabidopsis* roots treated with 50 nM metazachlor.

Our results indicate that metazachlor neither alters the total quantity of individual sterols, nor the quantity of FAs from glycerophospholipids, GlcCer or GIPCs pools (Fig. 2e). In addition, metazachlor treatment did not induce formation of tri-acyl-glycerol (TAG), indicating that our conditions did not create any redistribution of lipid metabolism from membrane lipids to storage lipids (Supplementary Fig. 2c). Moreover, metazachlor did not alter FAs composition of dicarboxylic acids and fatty alcohols compounds (Fig. 3a), which are known to be contained in the suberin polymer^35^. Instead, upon metazachlor we detected a strong decrease of h24:1, h24:0 and h26:0 in both GIPCs and GluCer pools (Fig. 2b,c). Decrease of non-hydroxylated 24:0 was also observed in the pool of FAs of glycerophospholipids, but non-hydroxylated 24:0 FAs represent only 2% of the pool of glycerophospholipids (Fig. 2a), whereas they represent more than 10% of the pool of GIPCs (Fig. 2c). Concomitantly, accumulation of hFAs, for which acyl chain length is under 24 carbons (≤h24), was observed on metazachlor treatment in pools of GlcCer and GIPC (Fig. 2b,c). Altogether, our results indicate that metazachlor is a unique tool to drastically invert the ratio between FAs≤24 and FAs≥24 within the pool of SLs (GlcCer and GIPCs) without decreasing their total quantity or significantly altering the pool of glycerophospholipids.

### Metazachlor alters root gravitropism

**Figure 4:**
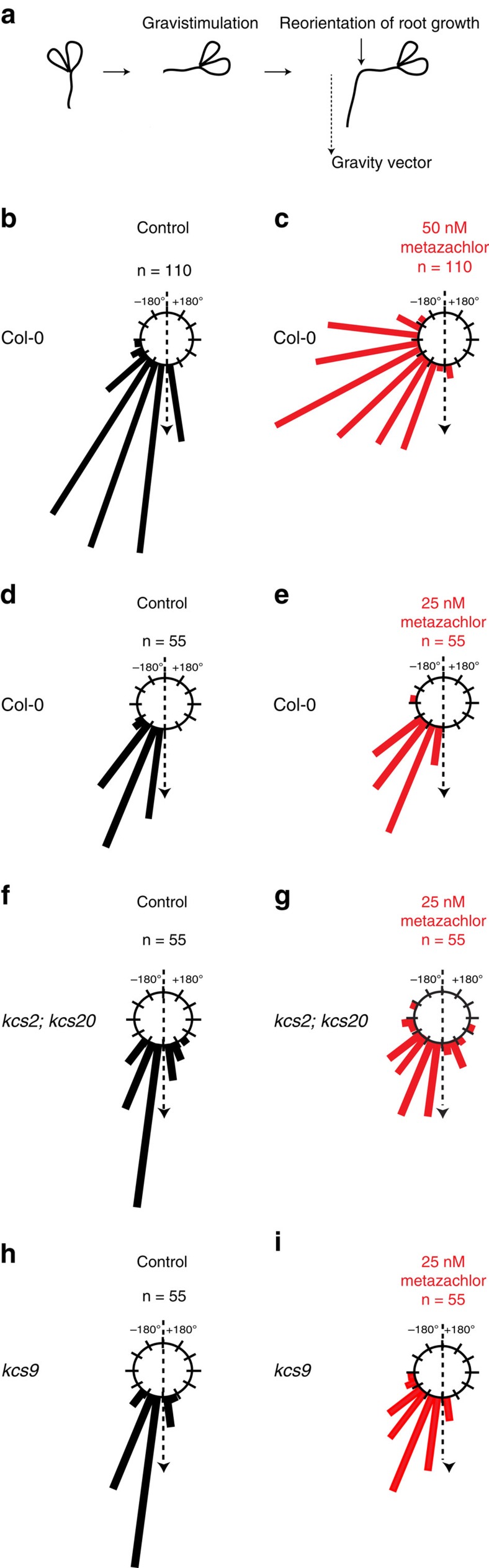
Metazachlor alters root gravitropism and targets KCS9 KCS2 and KCS20. (**a**) Root angle curvature towards the new gravity vector 24 h following a gravistimulation (turn the plate of 90°) is calculated, we then ranked the effective (n) into classes of 15° angles (0° was the exact direction of the new gravity vector) and represented each class of angles in a circular chart. (**b**) In untreated roots, reorientation of roots 24 h after a gravistimulation is very close to the gravity vector, whereas in 50 nM metazachlor-treated roots (**c**) this reorientation is much less efficient (*n*=110 roots per experiment). (**d**–**i**) As compared with untreated roots (**d**), the *kcs2,20* double mutant (**f**) and *kcs9* single mutant (**h**) do not display gravitropism phenotype. On 25 nM metazachlor treatment, *kcs2,20* double mutant (**g**) and *kcs9* single mutant (**i**) display obvious root gravitropism phenotype, whereas wild-type roots treated with 25 nM of metazachlor (**e**) do not react. Hence, *kcs2,20* double mutant (**f**,**g**) and *kcs9* single mutant (**h**,**i**) are hypersensitive to metazachlor in respect to root gravitropism (*n*=55 roots per experiment).

In *A. thaliana*, modulation of root growth axis in response to change in gravity (gravitropism) is known to rely on apical polarity of PIN2 in epidermal cells^6^,^15^,^16^. Hence, attenuation of gravitropic response provides a reliable readout for any defects in trafficking or polar localization of PIN2. To evaluate root gravitropism, we measured the angle formed between the root and the new gravity vector 24 h after a 90° turn (Fig. 4a). We ranked values into classes of 15° angles (0° was the exact direction of the new gravity vector) and represented every class of angles in a circular chart. Metazachlor treatment strongly altered the gravitropic response as measured by the reorientation of root growth axis after 24 h following gravistimulation (Fig. 4b,c and Supplementary Fig. 3a,b). Kinematic analyses showed that metazachlor-treated roots displayed very slight and slow response to change in the direction of gravity (Supplementary Fig. 3c). Importantly, 50 nM metazachlor did not strongly affect root length compared with mock-treated roots (Supplementary Fig. 3d). Thus, metazachlor effect on root graviresponse was not due to defect in root elongation *per se*.

### Metazachlor targets KCS enzymes during gravitropism

Inhibition of activity of several KCS enzymes by metazachlor was previously shown by expressing a number of *Arabidopsis* KCS (KCS1, KCS2, KCS5/CER60, KCS6/CER6, KCS17, KCS18/FAE1 and KCS20) in yeast^34^. As our study uses root gravitropism as a phenotypic readout for apical PIN2 polarity, we used publicly available micro-arrays data sets and previously published expression studies of KCS genes and found that among KCS enzymes targeted by metazachlor, KCS1, KCS2, KCS17 and KCS20 are highly expressed or expressed at medium level in primary roots^36^,^37^. Moreover, we also found that KCS4 and KCS9 are highly expressed or expressed at medium level in primary roots^36^,^37^. Previous studies have characterized reduction of C22- and C24-containing FAs accompanied by accumulation of C20-containing FAs in *kcs2,20* double knockout mutant and reduction of C24-containing FAs accompanied by accumulation of C20- and C22-containing FAs in *kcs9* knockout mutant^38^,^39^. Hence, we analysed root gravitropism of *kcs9* single mutant and *kcs2,20* double mutant.

The *kcs9* single mutant or the *kcs2,20* double mutant do not display obvious root gravitropism phenotype in mock condition (Fig. 4d,f,h). However, root gravitropism defect is clearly seen when *kcs9* single mutant and *kcs2,20* double mutant are treated with 25 nM metazachlor (Fig. 4g,i), whereas wild-type roots do not significantly display gravitropism defect on 25 nM metazachlor treatment (Fig. 4d,e). To correlate root gravitropism phenotype and VLCFAs content, we quantified the total pool of VLCFAs and show that VLCFAs level is significantly and similarly reduced in roots of either *kcs9* single mutant, *kcs2,20* double mutant or 25 nM metazachlor-treated wild-type roots as compared with untreated wild type (Supplementary Fig. 4a). However, this level is far from being as low as a 50 nM metazachlor treatment on wild-type roots (Supplementary Fig. 4a). Contrastingly, a 25 nM metazachlor treatment on *kcs9* single mutant or the *kcs2,20* double mutant reduces VLCFAs content to similar level of a 50 nM metazachlor treatment on wild-type roots (Supplementary Fig. 4a). These results show that *kcs9* and *kcs2,20* double mutant are hypersensitive to metazachlor with respect to VLCFAs level and graviresponse. This also suggests that KCS2, KCS20 and KCS9 are targets of metazachlor during root gravitropism, and that there is a threshold of VLCFAs quantity under which gravitropism defects are triggered. As we could not have the *kcs2,20,9* triple mutant available for this study, we cannot exclude that other KCS are also targeted by metazachlor during root gravitropism. However, root graviresponse of *kcs1*, *kcs4* or *kcs17* single mutants treated with 25 nM metazachlor is not different from their gravitropism phenotypes observed in mock conditions (Supplementary Fig. 4b–g). These results suggest that KCS1, KCS4 and KCS17 are not preferred targets of metazachlor in roots at the concentration used, although we cannot exclude the possibility that KCS1, 4 and 17 act redundantly and a triple mutant might be required to visualize an effect of a treatment with 25 nM metazachlor. Thus, our data suggests that KCS2, KCS20 and KCS9 are targeted by metazachlor in the roots. Interestingly, *kcs9* and *kcs2,20* double mutant have been shown to not only alter the level of VLCFAs and hVLCFAs but also the level of ω-hydroxylated-VLCFAs^38^,^39^. Global FAs analysis performed on *Arabidopsis* roots treated with 50 nM metazachlor also showed that 22:0 ω-OH and 24:0 ω-OH were reduced as compared with mock condition (Fig. 3a). To test whether 22:0 ω-OH and 24:0 ω-OH play a role in root gravitropism we used the *ralph/cyp86b1* mutant, which display almost complete absence of 22:0 ω-OH and 24:0 ω-OH in the root suberin without affecting the level of hFAs^40^ (Supplementary Fig. 5a,b). Our results show that *ralph/cyp86b1* mutant does not display root gravitropism phenotype and is not hypersensitive to 25 nM metazachlor treatment (Supplementary Fig. 5c,d). These results show that reduction of 22:0 ω-OH and 24:0 ω-OH does not attenuate gravitropism.

### PIN2 localization and polarity depends on VLCFAs of SLs

**Figure 5:**
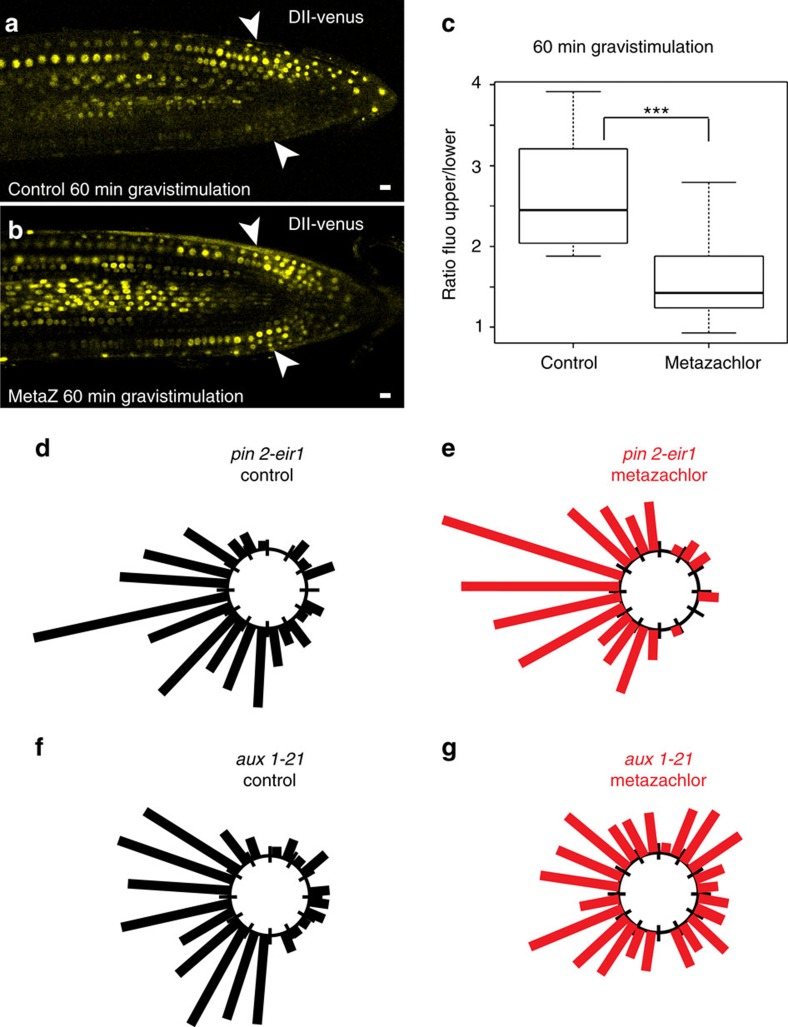
Metazachlor alters auxin distribution during root bending partly through the auxin efflux carrier PIN2. (**a**,**b**) Dynamic auxin redistribution 60 min after a gravistimulation, visualized by DII-venus fusion, shows differential auxin distribution in untreated roots (**a**) with a higher concentration of auxin at the upper side of the root (**a**), whereas metazachlor-treated roots do not display this differential repartition of auxin after gravistimulation. (**b**,**c**) Quantification of signal intensities between the upper and lower side of gravistimulated roots clearly results in a highly significant difference (*n*=10 seedlings for each experiment over 3 biological replicates). (**d**–**g**) The auxin efflux carrier mutant *pin2-*^*eir1*^ (**d**,**e**) and the auxin influx carrier mutant *aux1-21* (**f**,**g**) show similar phenotype in untreated roots (**d**,**f**) (*n*=110 root per genotype over 3 biological replicates). In metazachlor-treated roots, the *pin2-*^*eir1*^ mutant (**e**) appears more resistant to metazachlor than the *aux1-21* mutant (**g**) (*n*=110 roots per genotype over 3 biological replicates). Statistics were done by two-sided Welch's two sample *t*-test, ***P-value<0.001. All scale bars, 10 μm.

We next used the AUX/IAA auxin-interaction domain DII fused to the fluorescent protein Venus (DII-venus) to visualize dynamic changes in cellular auxin distribution^41^. In untreated roots, 1 h gravistimulation resulted in a much weaker DII-venus fluorescent signal at the lower side of the root compared with the upper side of the root (Fig. 5a). In contrast, no such reduction in DII-venus fluorescent signal was observed in metazachlor-treated roots on gravistimulation (Fig. 5b,c). These results show that SL composition is important for auxin redistribution and gravitropic response. Auxin redistribution during root gravitropism is known to rely on the auxin influx carrier AUX1 and the auxin efflux carrier PIN2 (refs 6, 15, 16, 42). Under control conditions, without metazachlor treatment, *aux1-21* or *pin2-eir1* mutants displayed similar response to gravistimulation (Fig. 5d,f). Interestingly, when the *aux1-21* mutant and the *pin2-eir1* mutant were subjected to metazachlor treatment, we could observe that metazachlor strongly enhances root gravitropism defect of the *aux1-21* mutant, while it only weakly enhanced *pin2-eir1* mutant phenotype (Fig. 5d–g). Although we cannot formally exclude the possibility that metazachlor treatment may target elements of gravitropic response in addition to PIN2, this appears less probable given the difference between *aux1-21* and *pin2-eir1* mutant response to metazachlor treatment. Importantly, we can conclude that modification of acyl-chain length of FAs by metazachlor targets a PIN2-mediated gravitropic response pathway rather than an AUX1-dependent pathway (Fig. 5d–g).

**Figure 6:**
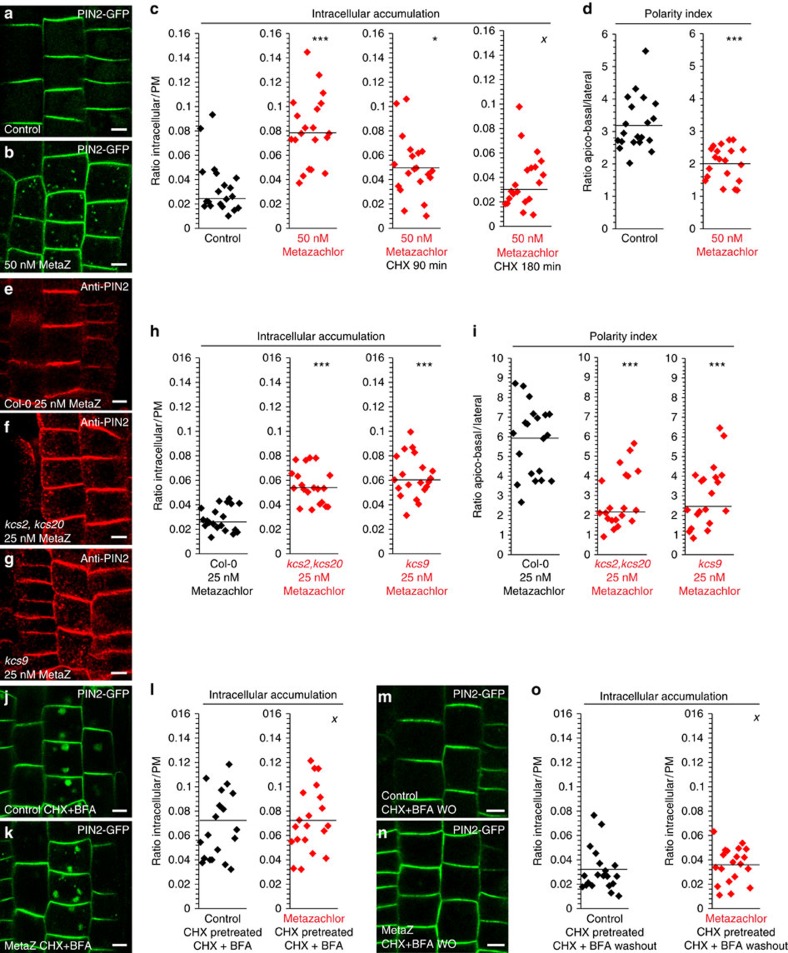
Reduction of VLCFAs alters apical polarity and secretory trafficking of PIN2 but not endocytosis and PM recycling of PIN2. (**a**–**c**) Compared with untreated cells (**a**), 50 nM metazachlor-treated cells (**b**) display intracellular accumulation of PIN2–GFP in endomembrane compartments. (**c**) Quantifications of fluorescence intensity ratios between the intracellular content and whole PM show a significant intracellular accumulation of PIN2–GFP in metazachlor-treated cells that is prevented by a pretreatment with 50 μM CHX from already 90 min pretreatment. (**d**) Quantifications of fluorescence intensity ratios between the apical-basal membranes and lateral membranes clearly indicate a significant loss of PIN2 polarity. (**e**–**i**) Compared with wild-type roots treated with 25 nM metazachlor (**e**) *kcs2,20* double mutant (**f**) and *kcs9* single mutant (**g**) display intracellular accumulation of anti-PIN2 (Alexa647) in endomembrane compartments and loss of PM polarity of PIN2. (**h**) Quantifications of fluorescence intensity ratios between the intracellular content and whole PM. (**i**) Quantifications of fluorescence intensity ratios between the apical-basal membranes and lateral membranes. (**j**–**l**) 50 μM BFA treatment, after a 50 μM CHX pretreatment, show no significant differences (**l**) in PIN2–GFP accumulation from the PM to the so-called intracellular ‘BFA bodies' between untreated cells (**j**,**l**) and metazachlor-treated cells (**k**,**l**), revealing that PIN2 endocytosis is not altered by metazachlor. (**m**–**o**) Washout of BFA in presence of CHX after a 50 μM CHX pretreatment and 50 μM BFA treatment show no significant differences (**o**) in PIN2 redistribution at PM from ‘BFA bodies' between untreated cells (**m**,**o**) and metazachlor-treated cells (**n**,**o**). Statistics were done by two-sided Wilcoxon's rank-sum test, ^X^*P*-value>0.05, *P-value<0.05, ***P-value<0.001, *n*=200 cells distributed over 20 roots for each experiment (3 biological replicates). All scale bars, 5 μm.

In agreement, we could not detect any changes in AUX1 localization on metazachlor treatment (Supplementary Fig. 6a,b). The ratio of fluorescence between the cell content and the whole PM (intracellular/PM) was identical for AUX1–YFP in metazachlor-treated roots compared with that in untreated roots (Supplementary Fig. 6c). Moreover, we did not detect any changes in localisation of PIN1 and PIN7 auxin efflux carriers, which are also involved in root gravitropism^43^,^44^ and which localize in a polar manner at the basal PM of cells (Supplementary Fig. 6d–l). PIN1–GFP either driven by its own promoter (pPIN1::PIN1–GFP) and expressed in vascular cells of roots (Supplementary Fig. 6d–f), or ectopically expressed in epidermal cells of roots using the promoter of PIN2 (pPIN2::PIN1-GFP2; Supplementary Fig. 6g–i), remained polarly localized at the basal membrane and did not display intracellular accumulation of PIN1–GFP on 50 nM metazachlor treatment (Supplementary Fig. 6d–i). Similarly, PIN7–GFP driven by its own promoter (pPIN7::PIN7–GFP) and expressed in vascular cells of roots also did not display localization defects when treated with 50 nM metazachlor (Supplementary Fig. 6j–l). In addition, in root epidermal cells the non-polar cargo SNARE protein NPSN12 fused to mCherry (Supplementary Fig. 6m–o) and the non-polar cargo aquaporin protein PIP1,4 fused to mCherry (Supplementary Fig. 6p–r) still localize at the PM and do not accumulate in intracellular compartments on 50 nM metazachlor treatment (Supplementary Fig. 6m–r). Contrastingly, in metazachlor-treated roots we observed a significant accumulation of the polar cargo PIN2–GFP in dotty structures and the ratio intracellular/PM was significantly higher compared with untreated roots (Fig. 6a–c). Accumulation of PIN2 in intracellular compartments was also detected in *kcs9* single mutant (Fig. 6e,g,h) and *kcs2,20* double mutant (Fig. 6e,f,h) treated with 25 nM metazachlor, the concentration at which graviresponse is significantly attenuated in these mutants compared with wild type (Fig. 4d–i). In contrast, we did not detect intracellular accumulation of PIN2 in mock-treated *kcs9* single mutant or *kcs2,20* double mutant (Supplementary Fig. 7a–d) in agreement with the absence of root gravitropism phenotype in mock-treated *kcs9* single mutant or *kcs2,20* double mutants (Fig. 4d,f,h). Next, we investigated whether metazachlor impinges on polar localization of PIN2 as well (Fig. 6a,b,d). The ratio between fluorescence at apico-basal PM and lateral PM was significantly reduced for PIN2–GFP on metazachlor treatment (Fig. 6d). Moreover, PIN2 polarity at PM was also altered in *kcs9* single mutant (Fig. 6e,g,i) and *kcs2,20* double mutant (Fig. 6e,f,i) treated with 25 nM metazachlor. Together, these results show that PIN2 polarity at apical PM and PIN2 secretory trafficking at TGN are dependent on FAs≥24-acyl-chain length of SLs at the TGN.

### VLCFAs of SLs are crucial for PIN2 secretory sorting at TGN

It has been shown earlier that TGN is a compartment where both secretory and endocytic/recycling pathways intersect^30^,^45^. Hence, accumulation of PIN2 in intracellular compartment might be due to either alteration of secretion or alteration of endocytosis/recycling. Strikingly, treatment with the protein biosynthesis inhibitor cycloheximide (CHX) nearly abolished PIN2–GFP accumulation in TGN/SYP61 compartments in wild-type seedlings treated with 50 nM metazachlor (Fig. 6c). These results strongly suggest that metazachlor blocks PIN2 secretory trafficking at TGN rather than PIN2 endocytosis at PM. However, it is still possible that attenuation of PIN2 polarity by metazachlor may be due to metazachlor targeting endocytosis/recycling at the TGN. Therefore, we next addressed whether metazachlor affects PIN2 endocytosis/recycling. To visualize PIN2 endocytosis, we pretreated seedlings with CHX to exclude visualization of *de novo* synthesized PIN2–GFP. Seedlings were next treated with CHX and BrefeldinA (BFA), which induces intracellular accumulation of endocytosed PIN2–GFP in so-called ‘BFA bodies'^46^,^47^ (Fig. 6j). We quantified that the ratio intracellular/PM increases identically between seedlings treated with metazachlor and metazachlor-free control (Fig. 6j–l). Moreover, when BFA was washed out, the ratio intracellular/PM decreased to the same level between metazachlor-treated and untreated seedlings (Fig. 6m–o). Previously, it has been shown that some types of SLs are involved in endocytosis and PM recycling of AUX1 and PIN1 potentially through RAB-A2a compartments that could then be considered as putative recycling endosomes^48^. Our results suggest that modification of acyl chain length of SLs has impacts on *de novo* PIN2 delivery to apical PM without perturbing PIN2 endocytosis or PIN2 recycling.

**Figure 7:**
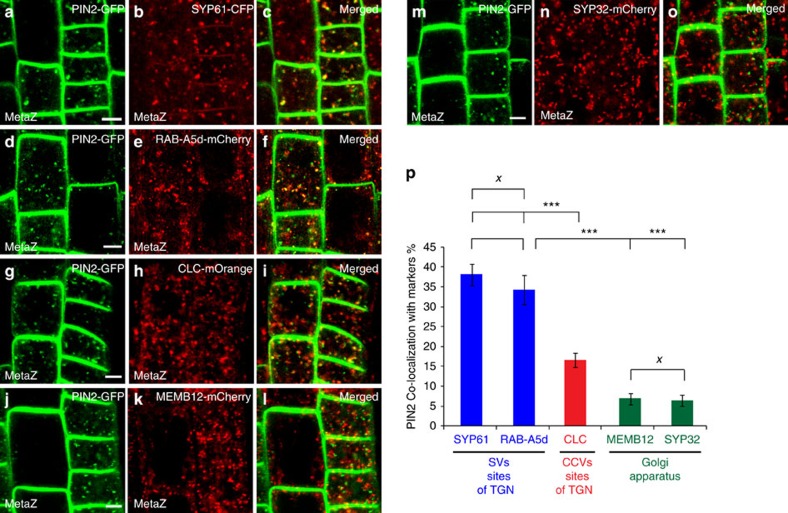
Metazachlor accumulates PIN2 at SVs sites of TGN. (**a**–**o**) Co-localization of endomembrane compartments labelled by PIN2–GFP (**a,d**,**g**,**j**,**m**) and either SYP61–CFP-labelled TGN-associated SVs (**b**), RAB-A5d-mCherry-labelled TGN-associated SVs (**e**), CLC-mOrange-labelled TGN-associated CCVs (**h**), MEMB12-mCherry-labelled Golgi apparatus (**k**) or SYP32-mCherry-labelled Golgi apparatus (**n**) on 50 nM metazachlor. (**c**,**f**,**i**,**l**,**o**) Merged pictures of corresponding pictures. (**g**) Quantification of co-localization events show a strong match of PIN2 with TGN-associated-SYP61/RAB-A5d-SVs, whereas weak co-localization is detected with MEMB12/SYP32-Golgi. (**p**) Co-localization values of SYP61/RAB-A5d with PIN2 and MEMB12/SYP32 with PIN2 are highly different. PIN2 co-localizes at medial level with TGN-associated-CLC-Clathrin vesicles but is significantly different from co-localization of PIN2 with SYP61/RAB-A5d. Statistics were done by two-sided Kruskal–Wallis rank sum test, ^X^*P*-value>0.05, ***P-value<0.001, *n*=40 cells distributed over 10 roots for each experiment (3 biological replicates). All scale bars, 5 μm. Errors bars are s.e.m.

Next, we identified which endomembrane compartment PIN2–GFP labelled on metazachlor treatment. Our results indicated that PIN2–GFP-labelled structures strongly co-localized with the TGN/SVs marker SYP61–CFP (Fig. 7a–c,p). PIN2–GFP-labelled structures also strongly co-localized with the RAB protein RAB-A5d fused to mCherry (Fig. 7d–f,p). Co-localization of RAB-A5d-mCherry with either the TGN/SVs marker ECHIDNA (ECH) or the TGN/CCVs CHC further indicated that RAB-A5d rather locates at SVs site of TGN (Supplementary Fig. 8a–c,g) rather than CCVs sites of TGN (Supplementary Fig. 8d–f,g). Hence, we could confirm with two independent markers that PIN2 accumulates at SVs sites of TGN on metazachlor. On metazachlor, only weak co-localization of PIN2 was observed with either the Golgi marker MEMB12 fused to mCherry (Fig. 7j–l,p) or with another Golgi marker, the syntaxin SYP32, fused to mCherry (Fig. 7m–o,p). Co-localization of PIN2–GFP-labelled structures and CLC-mOrange was higher than that observed for Golgi markers but much lower than what we observed for the two markers of SVs (Fig. 7g–I,p). Furthermore, metazachlor treatment did not abolish the separation of TGN from Golgi membranes as we did not detect a change in the amount of co-localization between the Golgi marker MEMB12–YFP with either the TGN/SVs marker ECH (Supplementary Fig. 1g–l,s) or the TGN/CCVs marker CHC (Supplementary Fig. 1m–r,s).

Altogether, these results suggest that on modification of acyl-chain length of SLs, PIN2 accumulates in a TGN subdomain labelled by SYP61 but not in a TGN subdomain labelled by clathrin or in the Golgi apparatus. These results are also consistent with the enrichment of hVLCFAs, which are almost exclusively contained in SLs (Fig. 2b,c), observed in immunoprecipitated SYP61 compartments compared with RAB-A2a or Golgi compartments observed in Fig. 1j,k.

### Metazachlor alters the morphology of SVs at TGN

**Figure 8:**
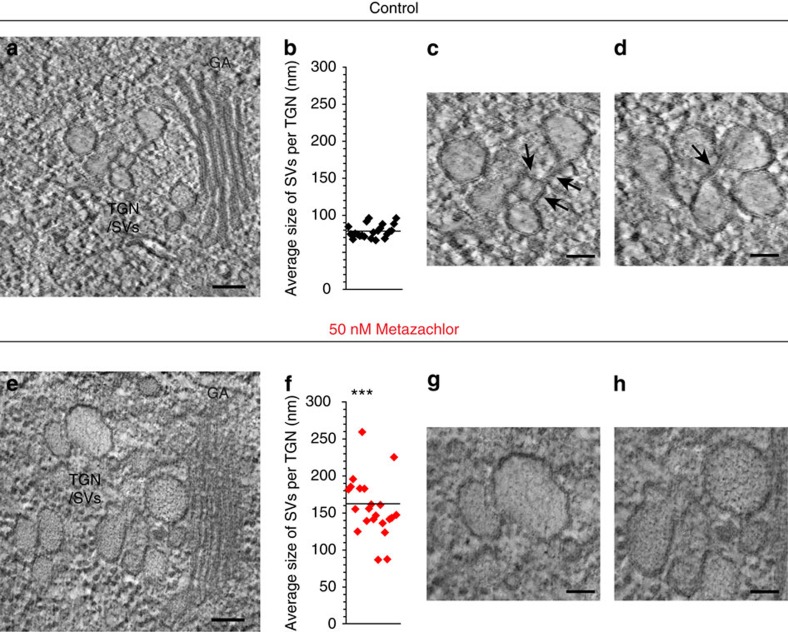
Metazachlor alters TGN-associated SVs morphology and tubular interconnections at TGN. Transmission electron microscopy (TEM) of TGN membrane structures in *Arabidopsis* root treated (**e**–**h**) or not (**a**–**d**) with metazachlor. (**a**) Untreated cell showing Golgi apparatus (GA) and the SVs visible as a tubulo-vesiculated membrane network (SVs/TGN) at the *trans*-side of the Golgi. (**e**) Metazachlor-treated cell (50 nM) showing Golgi apparatus and swollen TGN-associated SVs. (**b**,**f**) Quantification show that the average diameter of SVs per TGN is around 80 nm in untreated cells (**b**), while being around 160 nm in metazachlor-treated cells (*n*=22 TGN for each for each experiment over 3 biological replicates, statistics were done by two-sided Welch's two-sample *t*-test, ***P-value<0.001). (**f**,**c**,**d**) Magnification from **a** displaying tubular interconnections (black arrows) between SVs at TGN in untreated cells. (**g**,**h**) Magnification from **e** displaying larger SVs without tubular interconnections detected between them. Scale bars, 100 nm (**a**,**e**) and 50 nm (**c**,**d**,**g**,**h**).

Although TGN is still able to separate from Golgi apparatus in metazachlor-treated roots, we investigated whether metazachlor would alter TGN ultrastructure. Using high-pressure freezing and freeze substitution we could clearly see by transmission electron microscopy that alteration of acyl-chain length of SLs by metazachlor resulted in morphology alteration of TGN. We focused on elongating cells, which are more difficult to preserve than meristematic cells but in which we clearly saw PIN2 localization defects. Moreover, cells in the elongation zone of the root mediate differential cell growth during root gravitropism and allow the bending of the root^49^. In untreated cells, TGN appeared tubulo-vesiculated and SVs seemed to bud off from TGN tubules, while being progressively released (Fig. 8a). Contrastingly, in metazachlor-treated cells SVs of TGN appeared more swollen and seemed to remain in cluster (Fig. 8e). Quantification showed that in untreated cells the average diameter of SVs at TGN comprised between 60 and 100 nm (Fig. 8b). Contrastingly, average diameter of SVs in metazachlor-treated roots was rather comprised between 90 and 260 nm (Fig. 8f). Similar defects were also observed in roots chemically fixed with glutaraldehyde (Supplementary Fig. 9a,b), suggesting that enlarged diameter of SVs in metazachlor-treated roots were not due to potential ice crystals that could appear during the high-pressure freezing and freeze substitution procedure. In addition, we could observe in roots fixed by high-pressure freezing that although SVs were interconnected with tubules at TGN in untreated cells (Fig. 8c,d), metazachlor-treated cells hardly displayed SV-interconnecting tubules (Fig. 8g,h). These data show that acyl-chain length ≥24 of SLs is important for the machinery involved in regulating the size of SVs and the TGN membrane tubule network.

## Discussion

To date, distinct subdomains of TGN have been recognized based on non-overlapping localization of TGN proteins, for example, SYP61 and RAB-A2a in plants. Our data now reveal that subdomains of TGN are not only marked by distinct proteins but also display differential distribution of SLs. We demonstrate that differential enrichment of hFAs that contain acyl-chain length ≥24, almost exclusively present in SLs, are required for polar secretory sorting of apical-localized proteins, for example, PIN2 without affecting endocytosis or recycling. This is somehow reminiscent to what has been shown in yeast for the export from the endoplasmic reticulum (ER) of some glycosylphosphatidylinositol (GPI)-anchored proteins, which is sphingolipid dependent^50^. The explanation of this sorting was proposed to be based on the chemical/physical properties of SLs and GPI anchored proteins, which produce specific associations and subsequently constitute specific endoplasmic reticulum export sites where other proteins could be excluded^50^. Such specific SL–protein interactions were also recently demonstrated between a transmembrane protein and a C18-acyl-chain sphingomyelin species in animal cells^51^. In this study, we suggest that chemical/physical properties of acyl-chain length ≥24 of SLs govern PIN2 sorting at TGN. This could be achieved either by lipid–protein interactions as discussed above or by lipid–lipid interactions, or both. Indeed, at biochemical level it is well known that enrichment of SLs and sterols creates lateral auto-segregation of these lipids in micro-domains, for which the thickness and order of the bilayer is higher compared with the rest of the membrane^52^,^53^,^54^,^55^,^56^. It is hypothesized that these differential membrane properties aid in membrane sorting of proteins but underlying mechanisms are not known. Our study now shows that acyl-chain length ≥24 of SLs play a key role in apical delivery of secretory cargo from TGN. In model membranes, it has been shown that C24-containing SLs display a distinct phase behaviour and membrane packing as compared with C16-containing SLs, which are mostly phase separated^57^. Interestingly, C24-acyl chain of SLs can interdigitate with FAs of the opposing monolayer^58^. Therefore, interdigitation of acyl-chain length ≥24 of SLs could contribute to segregation of PIN2 at TGN. The observation that specific subdomains of TGN are enriched in VLCFA-containing SLs, and that perturbing this distribution leads to defects in TGN structure and function indicates that subcompartmentalization of TGN hinges on the nature and the length of the acyl chain of SLs. Indeed, phase separation induced by acyl-chain length of SLs is also directly linked to the shape of membranes, for example, vesicle versus tubule^59^. In addition, VLCFAs of SLs were shown to favour tubular structures due to their ability to form interdigitated phases^60^. Hence, concentration of VLCFA-containing SLs at the subdomain of the TGN where apical sorting occurs is fully consistent with the idea that phase separation at TGN is crucial to sorting. Our electron microscopy supports this hypothesis, as we observe less tubules versus vesicles when the length of acyl chains of SLs is shortened by metazachlor. Altogether, our results provide evidence for distinct SLs content of TGN subdomains and the importance of the length of acyl chains of SLs for polar sorting of proteins at TGN.

## Methods

### Plant material and growth conditions

The *A. thaliana* ecotype Colombia-0 (Col-0) and the following mutants were used: *pin2-eir1* (ref. 15), *aux1-21* (ref. 61), *kcs9* (ref. 38), *kcs2,20* (ref. 39), *kcs1* (GABI Kat GK-312G10), *kcs4* (SALK_095739C), *kcs 17* (GABI Kat GK-128C11) and *ralph/cyp86b1* (ref. 40). The following transgenic fluorescent protein marker lines in Col-0 were used: pRAB-A2a::YFP–RAB-A2a^20^, pSYP61::CFP–SYP61 (ref. 26), pUBQ10::YFP–MEMB12 (ref. 28), pUBQ10::mCherry-MEMB12 (ref. 28), pUBQ10::mCherry-RAB-A5d^28^, pUBQ10::mCherry-SYP32 (ref. 28), pUBQ10::mCherry-NPSN12 (ref. 28), pUBQ10::mCherry-PIP1;4 (ref. 28), p35s::DII-venus^41^, pPIN2::PIN2–GFP^62^, pPIN1::PIN1–GFP^12^, pPIN2::PIN1-GFP2 (ref. 13), pPIN7::PIN7–GFP^11^ and pAUX1-AUX1–YFP^63^. The p35s::CLC-mOrange fusion was in Wassilewskija background^64^. Seeds were sown on half Murashige and Skoog (MS) agar medium plates (0.8% plant agar, 1% sucrose and 2.5 mM morpholinoethanesulfonic acid (Sigma) pH 5.8 with KOH, left at 4 °C for 2 days and then grown in 16 h light/8 h darkness for 5 days before all experiments exception made for gravitropism assays (described hereafter) and when obtaining plant material for immunoprecipitation (described hereafter).

### Inhibitor treatments

For metazachlor (Greyhound Chromatography and Allied Chemicals) treatment, seedlings were grown on MS plates containing the drug at 50 nM in most experiments, except when specified. Metazachlor was added from a 100 mM stock in dimethylsulfoxide, an intermediate stock concentration at 100 μM was used extemporarily to make the plates. For cycloheximide (CHX) and BFA treatments, seedlings were treated in liquid medium (LM) containing 1 × MS, 1% sucrose, 2.5 mM morpholinoethanesulfonic acid pH 5.8. In BFA experiments, seedlings were first pretreated with 50 μM CHX (Sigma) for 90 min and then treated with 50 μM CHX and 50 μM BFA for 90 min. Washout experiments were performed by washing in LM implemented with 50 μM CHX for 90 min.

### Immunocytochemistry and confocal laser scanning microscopy

Whole-mount immunolabelling of *Arabidopsis* root was performed as described^65^. In brief, seedlings were fixed in 4% paraformaldehyde dissolved in MTSB (50 mM PIPES, 5 mM EGTA, 5 mM MgSO_4_ pH 7 with KOH) for 1 h at room temperature (RT) and washed three times with MTSB. Roots were cut on superfrost slides (Menzel Gläser, Germany) and dried at RT. Roots were then permeabilized with 2% Driselase (Sigma), dissolved in MTSB for 30 min at RT, rinsed four times with MTSB and treated with 10% dimethylsulfoxide+3% Igepal CA-630 (Sigma), and dissolved in MTSB for 1 h at RT. Aspecific sites were blocked with 5% normal donkey serum (NDS, Sigma) in MTSB for 1 h at RT. Primary antibodies, in 5% NDS/MTSB, were incubated overnight at 4 °C and then washed four times with MTSB. Secondary antibodies, in 5% NDS/MTSB, were incubated 1 h at RT and then washed four times with MTSB. Antibody dilutions were as follows: rabbit anti-CHC (Agrisera, AS10 690) 1/300, rabbit anti-PIN2 (ref. 66) 1/1,000; rabbit anti-MEMB11 (ref. 29) 1/300, TRITC-coupled donkey anti-rabbit IgG (Jackson Immunoresearch, 711-025-152) 1/300 and AlexaFluor 647 (A647)-coupled donkey anti-rabbit IgG (Jackson Immunoresearch, 711-605-152) 1/300. Confocal laser scanning microscopy was performed using Leica TCS SP5 AOBS and Leica TCS SP8 AOBS systems (Leica). For live-cell imaging, seedlings were mounted with LM medium between one 24 × 50 mm coverslip and one 24 × 24 mm coverslip separated with double-sided tape. Co-localization analyses were performed using geometrical object-based method^67^ and the JACoP plug-in of ImageJ^68^ (http://rsb.info.nih.gov/ij/plugins/track/jacop.html). Briefly, the distance between centroids of green-labelled and red-labelled objects was calculated for all possible combination. When the distance between two labelled structures is below the resolution limit of the objective (200 nm), the co-localization was considered as true. Laser excitation lines for the different fluorophores were 405 nm for 4,6-diamidino-2-phenylindole, 458 nm for CFP, 488 nm for GFP, 514 nm for YFP and venus, 561 nm for mCherry and TRITC, and 633 nm for A647. Fluorescence emissions were detected at 410–480 nm for 4,6-diamidino-2-phenylindole, 465–515 nm for CFP, 521–600 nm for GFP, YFP and venus, 566–650 nm for mCherry and TRITC, and 643–740 for A647. In multi-labelling acquisitions, detection was in sequential line-scanning mode with a line average of 4. An oil-corrected × 63 objective, numerical aperture=1.4 (HCX PL APO CS 63.0x1.40 OIL UV) was used in immunolabelling and live-cell imaging experiments.

### Immunoprecipitation of intact TGN and Golgi compartments

The method is based on previously published TGN immuno-isolation procedure with some modifications^25^. In brief, *Arabidopsis* seedlings are grown in 250 ml of LM in 500 ml flasks for 9 days under 120 r.p.m. shaking and 16 h light/8 h darkness cycle. Seedlings are transferred to a mortar pre-cooled on ice and then grinded with a pillar in three times more (w/v) vesicle isolation buffer: HEPES 50 mM pH 7.5, 0.45 M sucrose, 5 mM MgCl_2_, 1 mM dithiothreitol, 0.5% PVP (Sigma) and 1 mM phenylmethylsulfonyl fluoride. The homogenate is then filtered through a Miracloth mesh and centrifuged at 1,600 *g* for 20 min. The supernatant is transferred to a new tube and centrifuged two more times at 1,600 *g* for 20 min. Supernatant is then loaded on 38% sucrose cushion (the sucrose is dissolved in 50 mM HEPES pH 7.4) and centrifuged at 150,000 *g* for 3 h at 4 °C. The total pool of membranes is located at the interface between the sucrose and the supernatant. After removing the supernatant, a step-gradient sucrose is built on the top of the membrane interface with 33 and 8% sucrose solutions (dissolved in 50 mM HEPES pH 7.4) successively. Tubes are centrifuged overnight at 150,000 *g* at 4 °C. A band of membranes appears at the 33/8% sucrose interface and is harvested, diluted in 2–3 volume of 50 mM HEPES pH 7.4, centrifuged at 150,000 *g* for 2 h at 4 °C and resuspended in the resuspension buffer (50 mM HEPES pH 7.4, 0.25 M sucrose, 1.5 mM MgCl_2_, 150 mM NaCl, 1 mM phenylmethylsulfonyl fluoride and protease inhibitor cocktail from Sigma). This resuspended fraction is the TM fraction we used as input for the IPs. Immunoprecipitation was performed with magnetic Dynabeads coupled to proteinA according to the manufacturer's instructions (Invitrogen). For each IP, 150 μl of beads were first washed with PBS-Tween (137 nM NaCl, 2.7 nM KCl, 10 nM Na_2_HPO_4_, 1.8 nM KH_2_PO_4_ and 0.02% Tween-20), then incubated with 15 μl of rabbit anti-GFP antibodies (Invitrogen, A-11122) for 1 h with shaking at 4 °C. After one PBS-Tween wash, beads are equilibrated in the resuspension buffer for 10 min on ice. Beads bound the anti-GFP antibodies are then incubated with 1 ml of purified TM extract for 1 h with shaking at 4 °C. After incubation, eight washes are performed with 1 ml of resuspension buffer for 5 min with shaking at 4 °C for each wash. Beads bound to targeted vesicles are eventually resuspended in 50 μl of resuspension buffer.

### Western blottings of IP fractions

Polyacrylamide gels were casted using the TGX Stain-Free FastCast premixed acrylamide solution manufactured by Bio-Rad. After gel activation, proteins were visualized and imaged using a ChemiDoc MP imaging system (Bio-Rad). Initial step-gradient-purified TM fractions (IP input) and beads-IP fractions (IP output) were loaded at equal quantity on SDS–PAGE gel and subjecting to western blotting. To equally load TM fractions and IPs fractions, we quantified the whole individual tracks using ImageJ software and adjusted the quantity of proteins loaded in each track to reach equal loading. For western blotting, the following antibodies and dilutions were used: mouse anti-GFP recognizing CFP, GFP and YFP (Roche, 118144600001) 1/1,000, rabbit anti-Sec21p (Agrisera, AS08 327) 1/1,000, rabbit anti-Memb11 1/1,000 (ref. 29), rabbit anti-ECH^21^ 1/1,000, rabbit anti-SYP61 (ref. 69) 1/1,000, rabbit anti-V-ATPase (VHA-E) 1/2,000 (Agrisera, AS07 213), rabbit anti-PMA2 (ref. 70) 1/1,000 and rabbit anti-H^+^PM-ATPase (Agrisera, AS07 260) 1/1,000. Secondary antibodies were as follows: goat anti-mouse IgG-HRP conjugate (1/3,000, Bio-Rad, 1721011) and goat anti-rabbit IgG-HRP conjugate (1/5,000, Bio-Rad, 1706515). Pictures were acquired using a ChemiDoc MP imaging system (Bio-Rad). To calculate the IP efficiency, quantifications of intensities were done using the ImageJ software on pictures in which signals were white and background black. Boxes of exact same size were positioned on signals of the IP input line (TM fraction) and IP output (beads immunopurification) on anti-CFP/YFP blots. Uncropped pictures of full western blottings displaying enrichment of targeted compartments in IPs is available in Supplementary Fig. 2b.

### Characterization of lipid composition

For acyl-chain characterization of lipids from beads-IP fractions and global FAs analyses from roots, 25 μl of beads extracts or fresh roots were directly incubated with 1 ml of 5% sulfuric acid solution in methanol (implemented with standards: 5 μg ml^−1^ of C17:0 and 5 μg ml^−1^ of h14:0) for transesterification (exchange of the organic group of esterified/amidified FAs by the methyl group of methanol). Transesterification is made overnight at 85 °C and leads to production of FAs methyl esters (FAMEs). FAMEs are then extracted by adding 1 ml of NaCl 2.5% and 1 ml of hexane 99%. After vigorous shaking and centrifugation at 700 *g* for 5 min at RT, the higher phase is collected, placed in a new tube and buffered with 1 ml of 100 mM Tris, 0.09% NaCl pH 8 with HCl. After vigorous shaking and centrifugation at 700 *g* for 5 min at RT, the higher phase is collected, placed in a new tube and evaporated with needles evaporating pan. Then, 200 μl of *N*,*O*-Bis(trimethylsilyl)trifluoroacetamide+1% trimethylsilyl (BSTFA+1% TMCS, Sigma) were added and incubated at 110 °C for 20 min. After evaporation, FAMEs are resuspended in 100 μl of 99% hexane and run on GC-MS. For sterols characterization from beads-IP fractions, 25 μl of beads extracts were directly incubated with 1 ml chloroform/methanol (2:1) (implemented with the standard: 5 μg α-cholestanol) for 2 h at RT. Lipid extract was then washed with 1 ml 0.9% NaCl, vigorously shaked and centrifuged at 700 *g* for 5 min at RT. The organic (lower) phase is collected and evaporated. Then, a saponification is performed on the lipid extract by incubating with 1 ml 99% ethanol and 100 μl of 11 N KOH for 1 h at 80 °C. After incubation, 1 ml of 99% hexane and 2 ml of water are added. After vigorous shaking and centrifugation at 700 *g* for 5 min at RT, the higher phase is collected, placed in a new tube and buffered with 1 ml of 100 mM Tris, 0.09% NaCl pH 8 with HCl. After evaporation, sterols are incubated with 200 μl BSTFA+1% TMCS, at 110 °C for 20 min. After evaporation, sterols are resuspended in 100 μl of 99% hexane and run on GC-MS.

For lipid characterization in *Arabidopsis* roots, lipids were extracted using methyl-*tert*-butyl ether (MTBE) as described previously^71^ and separated by high-performance thin-layer-chromatography (HPTLC). In brief, *Arabidopsis* roots were collected and incubated in 1 ml of boiling isopropanol for 10 min. Samples were then grinded in 5 ml of MTBE/methanol/water (10/3/2.5) in a glass potter and then transferred into a glass tube (called A). Samples were then heated at 60 °C for 30 min. Next, 3–4 ml of NaCl 0.9% was added to tube A and, after a vigorous shaking, was incubated another time at 60 °C for 30 min and then centrifuged at 700 *g* for 5 min at RT. The upper phase of tube A was collected and transferred into a new tube (called B). To the lower phase of the tube A, 3–4 ml of 100% MTBE was added. After shaking and centrifugation at 700 *g* for 5 min at RT, the upper phase of tube A was collected and added to tube B where the upper phase was saved previously. After evaporation of tube B, lipids were resuspended in chloroform/methanol/water (3/6/0.8).

For GlcCer separation, lipids were separated by HPTLC using the following migration solvent: methyl acetate, *n*-propanol/chloroform/methanol/0.25% KCl (2.5/2.5/2.5/1/0.9). For GIPCs, MTBE-dried extract was first de-esterified (to remove glycerophospholipids) by dissolving in 2 ml of 33% methylamine solution in ethanol/water (7:3 v/v) and incubating at 50 °C for 1 h. After hydrolysis the sample was dried and dissolved with heating and gentle sonication in chloroform/methanol/water (3/6/0.8). Then, lipids were separated by HPTLC impregnated with freshly prepared 0.2 M ammonium acetate dissolved in methanol. The migration solvent for GIPCs migration was as following: chloroform/methanol/NH_4_OH 4 N (in water) (9/7/2). Following this migration, glycerophospholipids were collected at the top of the plate and GIPCs were collected at the bottom of the plate. For both GlcCer and GIPCs separations, plates were run emptied with respective migration solvent before the loading of the plate. For both GlcCer and GIPCs, lipids on plate were stained with primuline. Pictures were acquired using a ChemiDoc MP imaging system (Bio-Rad). GlcCer and GIPCs spots were scratched off from plates and used for further analyses for acyl-chain composition using the above described procedure to produce FAMEs followed by GC-MS analyses.

We always normalized non-hydroxylated FAs using heptadecanoic acid (17:0) and normalized hydroxylated FAs using 2-hydroxyltetradecanoic acid (h14:0). As GC-MS is measuring a mass of compound and not a number of molecules, we used the molecular weight of each individual FAs (that were transesterified to volatized FAs in GC and resulted in FAMEs) to calculate the FAs content, expressed either as nmol% (when compared to the total pool of FAs) or nmol mg^−1^ fresh weight (when compared with the weight of starting material.

To quantify TAGs and DAGs lipids, roots from 5-day-old seedlings were grinded and homogenized in 1 ml of CHCl3-MeOH (2/1) in sintered glass tubes. Lipid extracts were then washed three times by an aqueous solution containing 0.9% of NaCl. After solvent evaporation, lipid extracts were resuspended in 100 μl of CHCl3-MeOH (1/1). TAGs were separated from other lipids on HPTLC silica plates (silica gel 60 F 254, Merck, Germany) eluted with Hexane/Diethylether/acetic acid (90/15/2). Identification of lipids was done using lipid standards from Aventi Lipids (USA) and lipid quantification was performed by densitometry analysis^72^ using a TLC scanner 3 (CAMAG). The amounts (μg) of lipids were determined by using standard curves established with the standard lipids.

### Root gravitropism assays and DII-venus visualization

Seedlings were grown on agar plates, cultured vertically at 22 °C under a 16 h light/8 h dark cycle for 3 days. They were then transferred to darkness under the same growth condition and incubated for a further 24 h, maintaining the same growth plate orientation. Next, plates were turned counter-clockwise through 90° and incubated vertically in the dark for 24 h under the same growth conditions. Photographs were then taken and the angle formed between the root tip and the new gravity vector was measured using ImageJ Software. For kinematic analyses seedlings were photographed starting from the gravistimulation at 1 h intervals for 24 h using a Canon D50 without infrared filter, remotely controlled by Canon Remote. The angles were ranked into twelve 15° negative values classes (from −180 to 0) and into twelve 15° positive values classes (from 1 to 180). Percentage from the total number of roots angles measurements was charted per class. Data presented in gravitropism charts were pooled from three independent experiments.

For DII-venus visualization, live imaging was performed 60 min after gravistimulation. Fluorescence at the root tip was acquired using strictly identical acquisition parameters (laser power, photomultiplier, offset, zoom factor and resolution) between the treated and untreated line. Fluorescence intensity was measured on the two opposite sides of the root using LAS AF Lite Software. Background fluorescence was subtracted. Finally ratio between upper side and lower side was calculated.

### Transmission electron microscopy

High-pressure freezing was performed on root of 5-day-old plants with a LEICA EM-PACT1 device. To easily separate the frozen samples of the high-pressure freezing carrier, the supports were previously coated with 2% phosphatidycholine. As cryoprotectant we used a solution of 20% BSA diluted in LM implemented or not with 50 nM metazachlor. Freeze-substitution steps were achieved in a LEICA AFS2 system as follows: −90 °C during 72 h in acetone with 2% OsO_4_ and 0.1% uranyl acetate, the temperature was then increased at the rate of 3 °C h^−1^ until −50 °C was reached. Then, washings in acetone followed by washings in ethanol were performed. The embedding step was progressively achieved in the lowicryl resin HM20 (EMS) at −50 °C before the resin was polymerized under ultraviolet during 48 h at −50 °C followed by 48 h at 20 °C.

Chemical fixation of roots was performed on 5-day-old *Arabidopsis* roots that were fixed for 1 h in paraformaldehyde 1% + glutaraldehyde 3% dissolved in 0.1 M cacodylate buffer (pH 7.2), rinsed, incubated with tannic acid (0,1% in water) for 30 min, rinsed, postfixed in 1% OsO_4_ in phosphate buffer 1 h, rinsed, dehydrated through an ethanol series and impregnated in increasing concentrations of SPURR^73^ resin over a period of 2 days before being polymerized at 70 °C for 19 h.

For transmission electron microscopy observations, ultrathin sections of 70 nm thicknesses were made and imaged using a transmission electron microscopy FEI Tecnai G2 Spirit TWIN 120kV equipped with a CCD 16Mpixels Eagle 4 k.

### Statistics

All data analysed were unpaired (samples independent from each other). Normal distribution (Gaussian distribution) of data set was tested using Shapiro–Wilk normality test. On data normally distributed, sample homoscedasticity was assessed using Bartlett test before performing parametric tests. On data that were not normally distributed (or on data sets for which *n* <10), non-parametric tests were performed. To compare two data sets, Welch two sample *t*-test was performed on data set normally distributed, whereas Mann–Whitney test was used as non-parametric test. To compare multiple data sets, Kruskal–Wallis test was used as non-parametric test. Tukey's test was used as a single-step multiple comparison procedure to find means significantly different from each other. All statistical tests were two-tailed (two-sided test). All statistical analyses were performed with R i386 3.1.0 software. *P*-values were as follows: ^X^*P*-value>0.05 (nonsignificant), *P<0.05, **P<0.01 and ***P<0.001. Variances between each group of data were either represented in box plot or by the s.d. Sample sizes to ensure adequate power were as follows: co-localization experiments *n*=at least 40, lipid analyses on immunoprecipitated compartments *n*=at least 10, lipid analyses on *Arabidopsis* roots *n*=at least 4, root gravitropism assays *n*=at least 50, single localization in *Arabidopsis* roots *n*=at least 20 roots.

### Data availability

The authors declare that all data supporting the findings of this study are available within the article and its Supplementary Information files or are available from the corresponding author on request.

## Additional information

**How to cite this article:** Wattelet-Boyer, V. *et al*. Enrichment of hydroxylated C24- and C26-acyl-chain sphingolipids mediates PIN2 apical sorting at *trans*-Golgi network subdomains. *Nat. Commun.* 7:12788 doi: 10.1038/ncomms12788 (2016).

## Supplementary Material

Supplementary Information Supplementary Figures 1 - 9
