# Supplementary material for: Enrichment of hydroxylated C24- and C26-acyl-chain sphingolipids mediates PIN2 apical sorting at trans-Golgi network subdomains
Source: Nat Commun. 2016 Sep 29;7:12788. doi: 10.1038/ncomms12788 (PMC5056404; doi:10.1038/ncomms12788)
Supplement: Supplementary Information — Supplementary Figures 1 - 9 [file ncomms12788-s1.pdf]

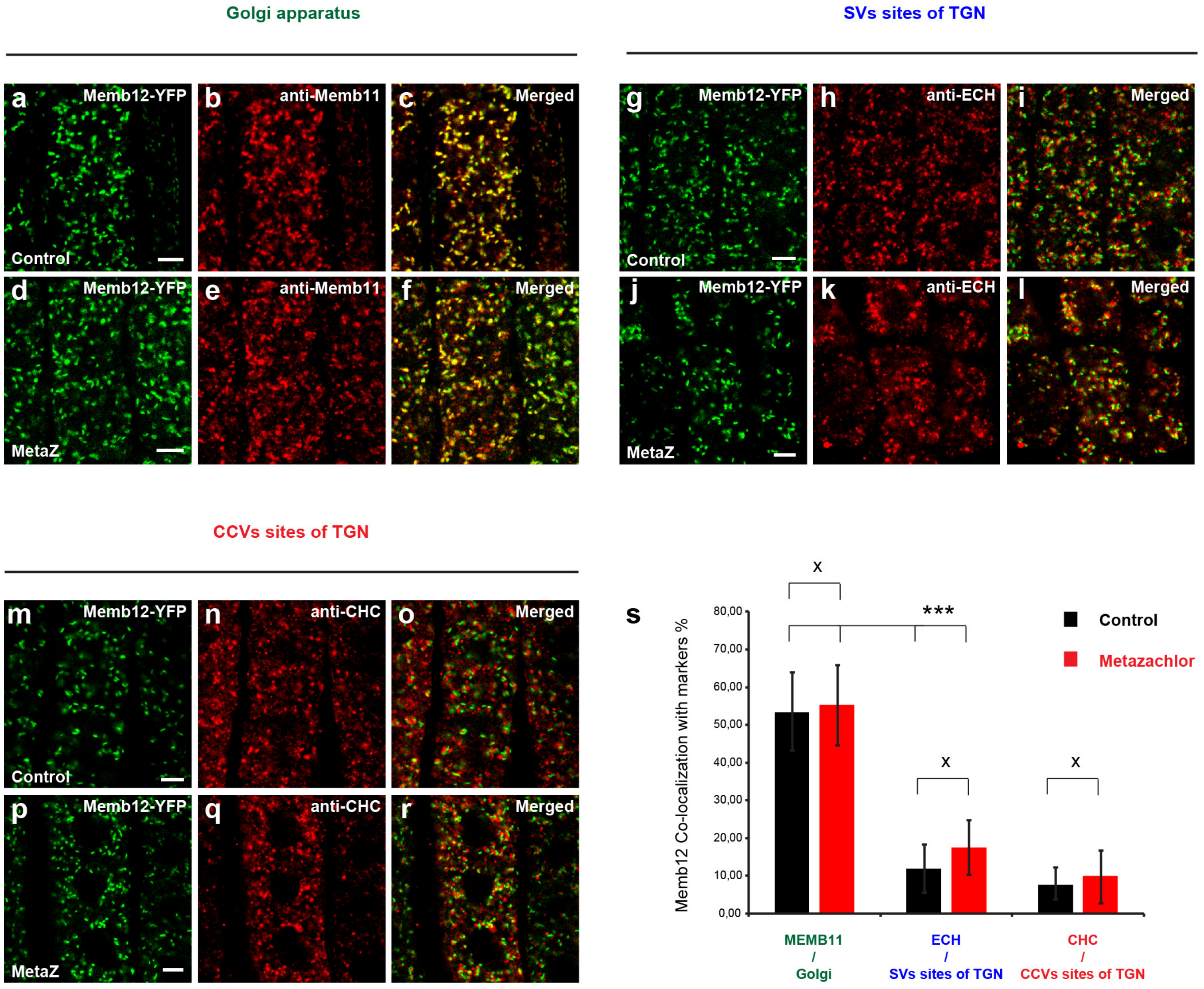

Supplementary Fig. 1. The Golgi marker MEMB12 is distinct from the SVs/TGN marker ECH and the CCVs/TGN marker CHC and this separation is not abolished by metazachlor treatment. (**a-f**) The Golgi marker MEMB12-YFP (**a, d**) strongly co-localises with the Golgi marker MEMB11 (**b, e**) both in mock treated (**a-c**) and metazachlor-treated (**d-f**) roots. (**c, f**) merged pictures of **a, b** and **d, e** respectively. (**g-l**) The Golgi marker MEMB12-YFP (**g, j**) is distinct from the SVs/TGN marker ECH (**h, k**) both in mock treated (**g-i**) and metazachlor-treated (**j-l**) roots. (**i, l**) merged pictures of **g, h** and **j, k** respectively. (**m-r**) The Golgi marker MEMB12-YFP (**m, p**) is distinct from the CCVs/TGN marker CHC (**n, q**) both in mock treated (**m-o**) and metazachlor-treated (**p-r**) roots. (**o, r**) merged pictures of **m, n** and **p, q** respectively. (**s**) Quantification of co-localisation between the Golgi marker MEMB12-YFP and either MEMB11 (Golgi), ECH (SVs/TGN) or CHC (CCVs/TGN). Statistics were done by two-sided Kruskal-Wallis rank sum test, X p-value > 0.05, *** p-value < 0.001, n = 40 cells distributed over 10 roots for each experiment (3 biological replicates). All scale bars are 5 µm. Errors bars are s.e.m.

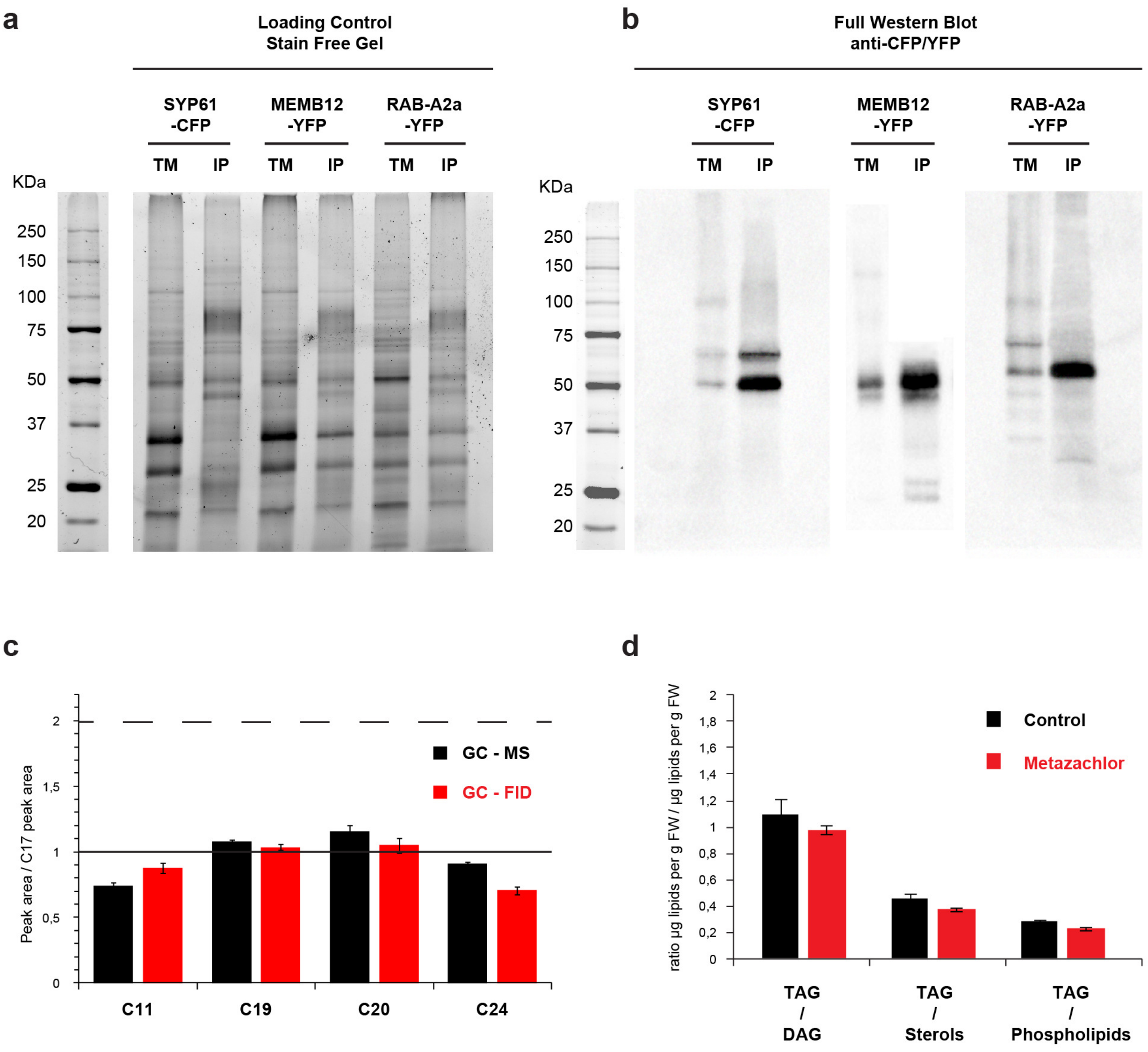

Supplementary Fig. 2. (**a**) Loading control of Fig. 1. Stain-Free polyacrylamide gel of total membrane fractions (TM: IP input) and immuno-precipitated fractions (IP: IP output) of SYP61-CFP (SVs/TGN), MEMB12-YFP (Golgi) and RAB-A2A-YFP (CCVs/TGN) compartments. Western-blots of corresponding gel are displayed in Fig. 1i. (**b**) Uncropped western-blots of anti-CFP/YFP blots in Fig. 1i showing enrichment of targetted compartments in IPs output (IP) as compared to IPs input (TM). (**c**) GC-MS and GC-FID analyses of fatty acids with different acyl-chain length (C11, C19, C20 and C24) loaded at the same quantity (50 µg) and showing similar results between GC-MS and GC-FID. Areas of peaks were integrated and normalized to the area of an internal standard (heptadecanoic acid, 17:0) added at the same amount (50 µg). (**d**) Triacylglycerol (TAG) and diacylglycerol (DAG) quantification in roots of 5-day-old Arabidopsis seedlings show no differences between untreated (black) and metazachlor-treated roots (red), whether for the TAG/DAG ratio or for the TAG/sterols or TAG/phospholipids ratios. Errors bars are s.d.

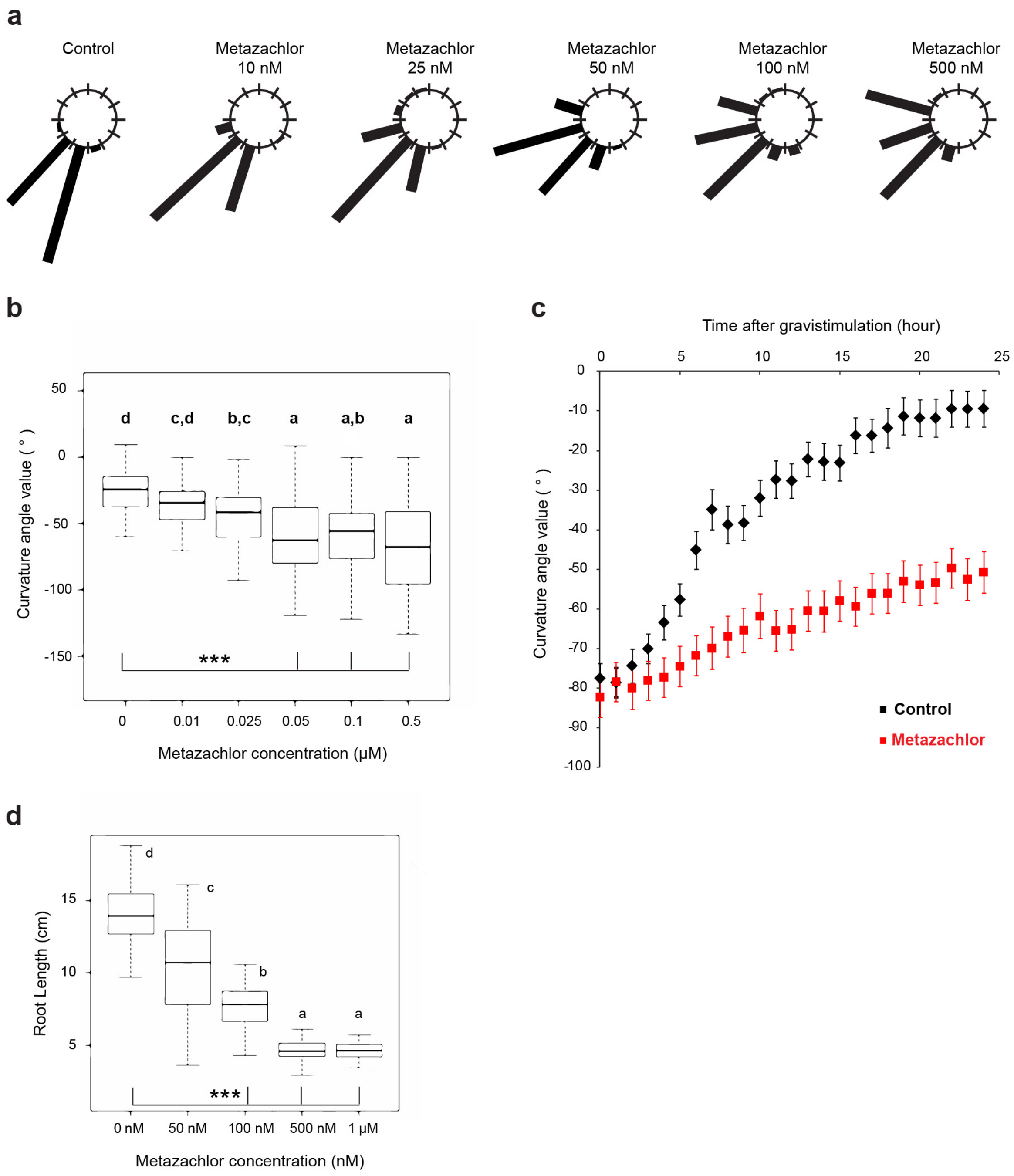

Supplementary Fig. 3. Differential growth rather than the actual axial growth is altered by metazachlor during root gravitropism. (**a**) Dose-response effect of metazachlor on the repartition of root angles bending 24h after gravistimulation. (**b**) Statistical analyses confirmed that 50 nm is the first concentration of metazachlor to produce an effect on root gravitropism (n = 72 seedlings per concentration over 3 biological replicates). (**c**) Kinematic analyses of root angles bending hour by hour following the gravistimulation (n = 24 seedlings per experiment over 2 biological replicates). Compared to untreated roots (black curve), metazachlor-treated roots (red curve) display root bending defects already from 3 hours after gravistimulation and a very slow bending towards the new gravity vector. (**d**) Statistical analyses confirmed that 50 nM metazachlor treatment results in root length close to that of untreated roots while higher concentration strongly reduce root length (n = 60 seedlings per concentration over 3 biological replicates). Statistics were done by two-sided Kruskal-Wallis rank sum test, *** p-value < 0.001.

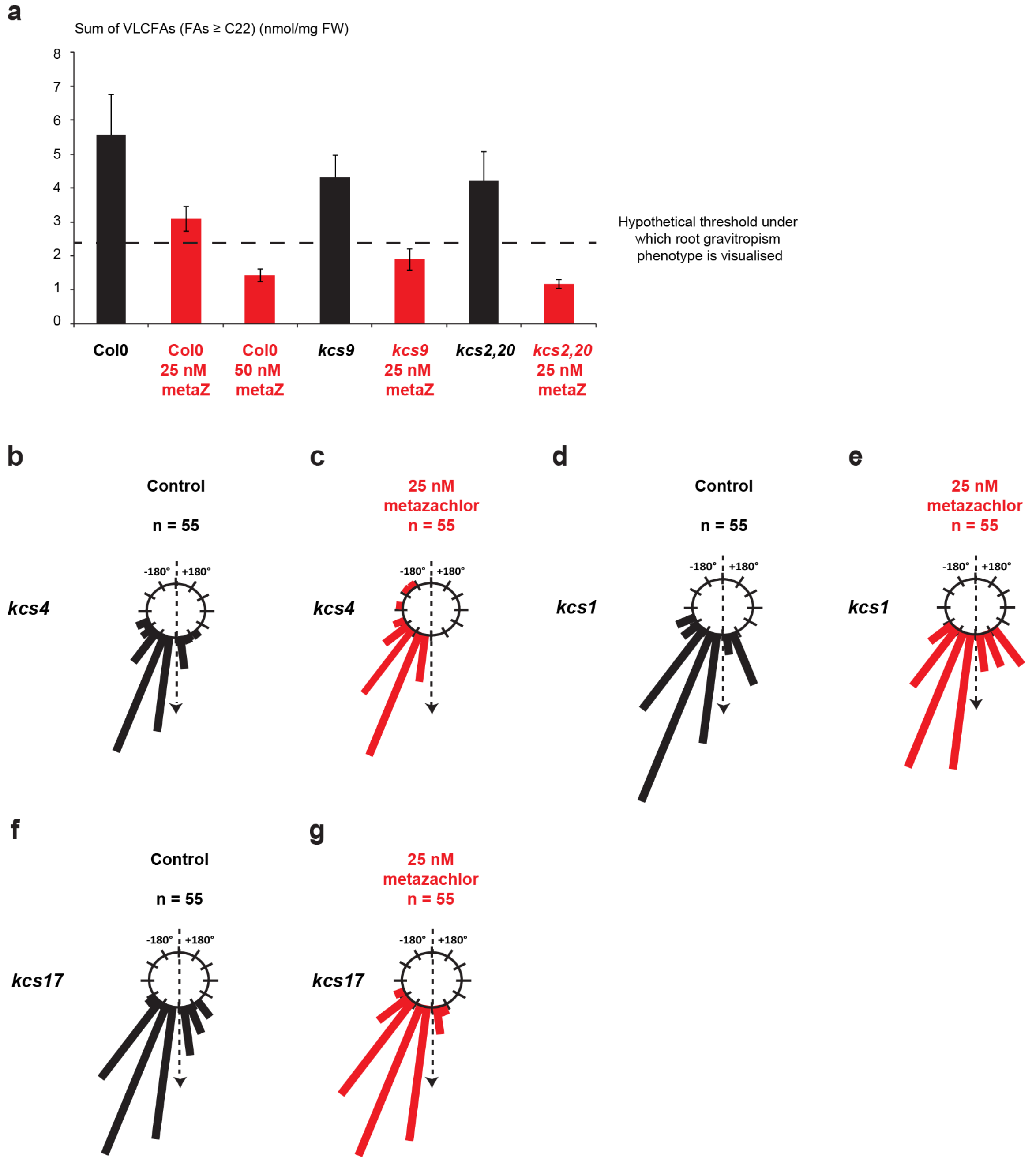

Supplementary Fig. 4. (a) Sum of VLCFAs (nmol of VLCFAs/mg Fresh Weight, VLCFAs are FAs ≥ C22) in wild-type roots (Col0) treated with 25 nM or 50 nM metazachlor and in *kcs9* and *kcs2,20* mutants roots treated with 25 nM metazachlor. Wild-type roots (Col0) show a progressive decrease of VLCFAs upon increasing concentration (25 nM and 50 nM) of metazachlor. *kcs9* or *kcs2,20* mutants show a decrease of VLCFAs, as compared to wild-type, but not as much as 50 nM treatment with metazachlor on wild-type roots. While wild-type roots treated with 25 nM metazachlor show an intermediate decrease of VLCFAs, *kcs9* or *kcs2,20* mutants treated with 25 nM metazachlor display a strong decrease of VLCFAs, equivalent to a 50 nM metazachlor treatment on wild-type roots. n = 4 for each experiment, 4 biological replicates. Errors bars are s.d. **(b-g) Root gravitropism is not mediated by the individual action of either KCS4, KCS1 or KCS17.** Root gravitropism assays on *kcs4* mutant (**b**, **c**), *kcs1* mutant (**d**, **e**) and *kcs17* mutant (**f**, **g**) yield to similar results between untreated roots (**b**, **d**, **f**) and roots treated with 25 nM of metazachlor (**c**, **e**, **g**) suggesting that metazachlor effect on root gravitropism is not mediated by the individual action of KCS4, KCS1 or KCS17. Statistics were done by two-sided Kruskal-Wallis rank sum test, *** p-value < 0.001.

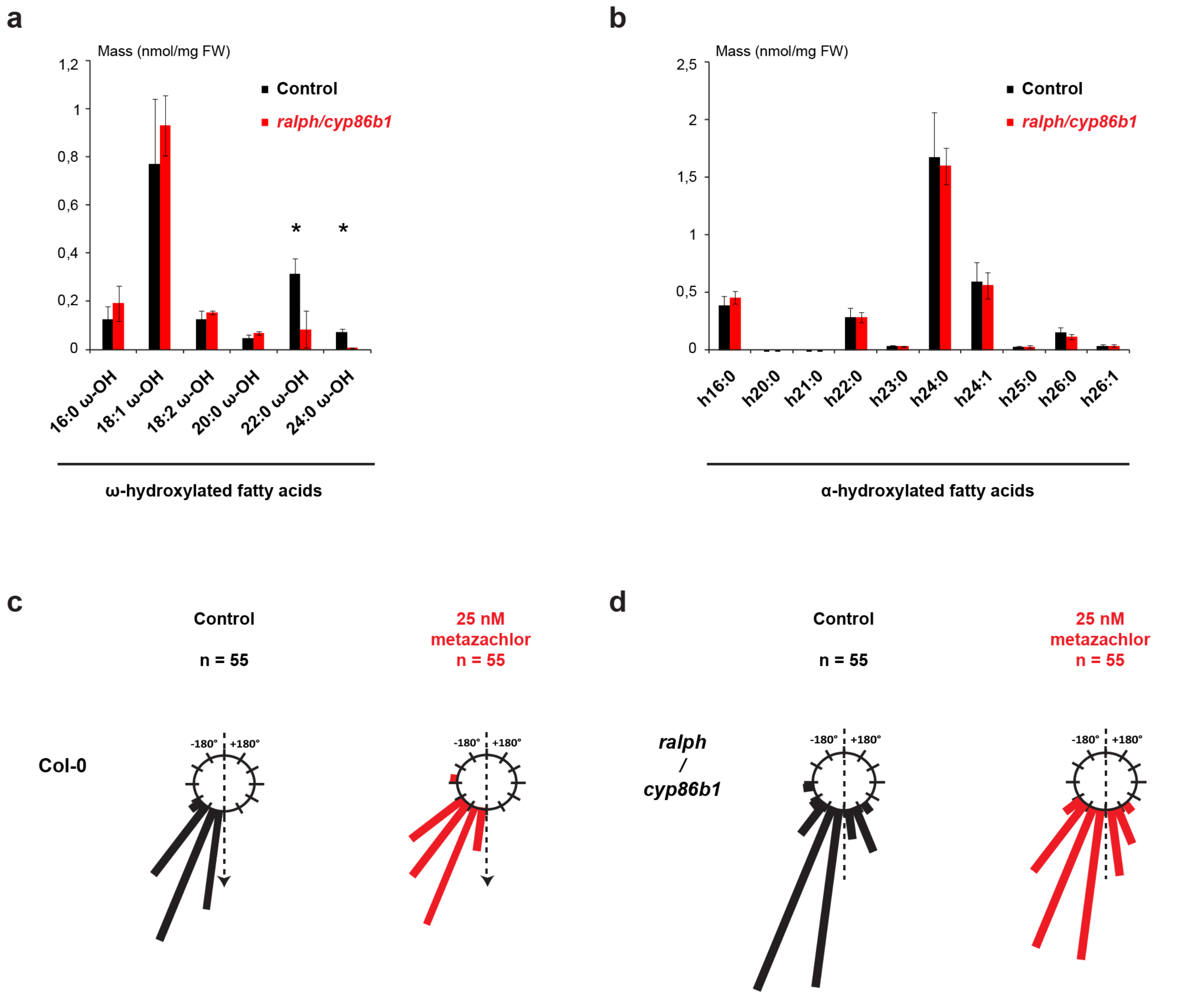

Supplementary Fig. 5. The *ralph/cyp86b1* mutant has reduced ω-hydroxylated VLCFAs but is not altered in α-hydroxylated VLCFAs and neither display root gravitropism phenotype nor hypersensivity to metazachlor. (**a**, **b**) Global lipid analysis of the *ralph/cyp86b1* mutant shows decrease of ω-hydroxylated VLCFAs (**a**) but reveals no alteration of α-hydroxylated VLCFAs (n = 4 for each genotype) (**b**). (**c**) Root gravitropism assay of untreated wild-type (Col-0) (black) and wild-type treated with 25 nM metazachlor (red). (**d**) Root gravitropism assay of untreated *ralph/cyp86b1* mutant (black) and *ralph/cyp86b1* mutant treated with 25 nM metazachlor (red). No significant differences could be detected between untreated Col-0 and 25 nM metazachlor-treated Col-0 and between untreated *ralph/cyp86b1* mutant and 25 nM metazachlor-treated *ralph/cyp86b1* mutant (n = 55 for each experiment). Statistics were done by two-sided Wilcoxon rank sum test, * p-value < 0.05, n = 4 for each experiment, 4 biological replicates. Errors bars are s.d.

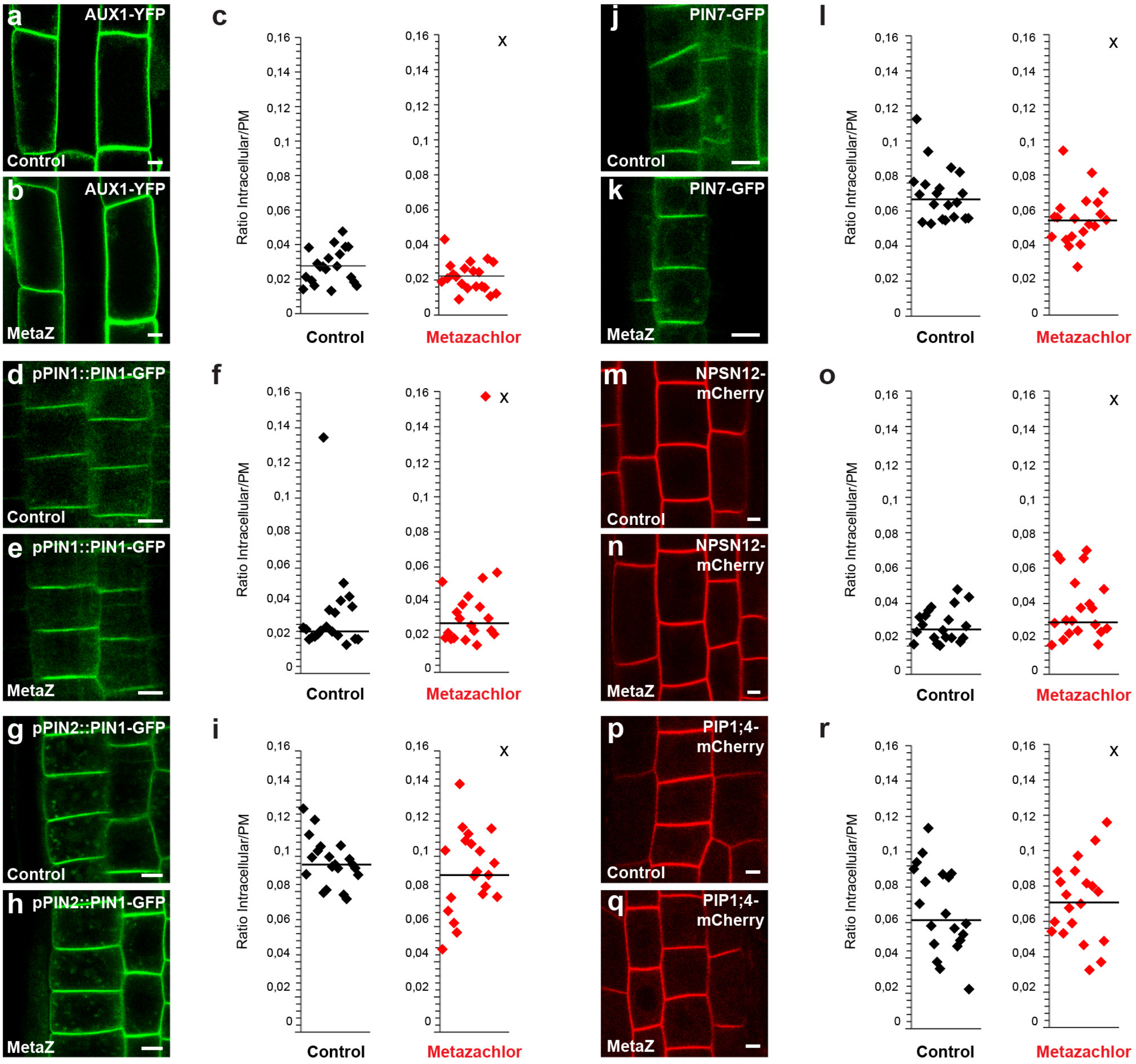

Supplementary Fig. 6. Metazachlor neither alters localization of AUX1, PIN1 and PIN7 auxin carriers nor localization of the non-polar cargos NPSN12 and PIP1;4. (**a**, **b**) Subcellular localization of AUX1-YFP, driven by its own promotor, in epithelial cells of untreated roots (**a**) and 50 nM metazachlor-treated roots (**b**). (**d**, **e**) Subcellular localization PIN1-GFP, driven by its own promotor, in vascular cells of untreated roots (**d**) and 50 nM metazachlor-treated roots (**e**). (**g**, **h**) Subcellular localization PIN1-GFP, driven by the PIN2 promotor, in epithelial cells of untreated roots (**g**) and 50 nM metazachlor-treated roots (**h**). (**j**, **k**) Subcellular localization PIN7-GFP, driven by its own promotor, in vascular cells of untreated roots (**j**) and 50 nM metazachlor-treated roots (**k**). (**m**, **n**) Subcellular localization NPSN12-mCherry, driven by the constitutive UBQ10 promotor, in epithelial cells of untreated roots (**m**) and 50 nM metazachlor-treated roots (**n**). (**p**, **q**) Subcellular localization of PIP1;4, driven by the constitutive UBQ10 promotor, in epithelial cells of untreated roots (**p**) and 50 nM metazachlor-treated roots (**q**). (**c**, **f**, **i**, **l**, **o**, **r**) Quantifications of fluorescence intensity ratios between the intracellular content and whole plasma membrane show no significant differences between untreated roots and 50 nM metazachlor-treated roots for all markers tested (n = 200 cells distributed over 20 roots for each experiment, 3 biological replicates). Statistics were done by two-sided Wilcoxon rank sum test, X p-value > 0.05. All scale bars are 5 µm.

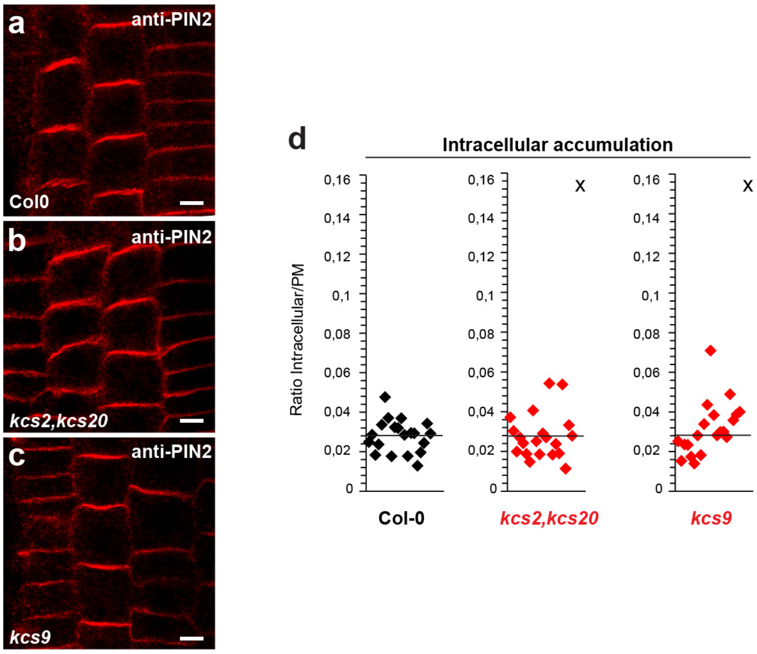

Supplementary Fig. 7. PIN2 localization is not altered in *kcs2,20* double mutant or *kcs9* single mutant untreated with metazachlor. (**a-c**) Compare to wild-type roots (**a**) *kcs2,20* double mutant (**b**) and *kcs9* single mutant (**c**) display similar localization of anti-PIN2 (Alexa647) in epithelial root cells. (**d**) Quantifications of fluorescence intensity ratios between the intracellular content and whole plasma membrane, n = 200 cells distributed over 20 roots for each experiment, 3 biological replicates). Statistics were done by two-sided Kruskal-Wallis rank sum test, X p-value > 0.05. All scale bars are 5 µm.

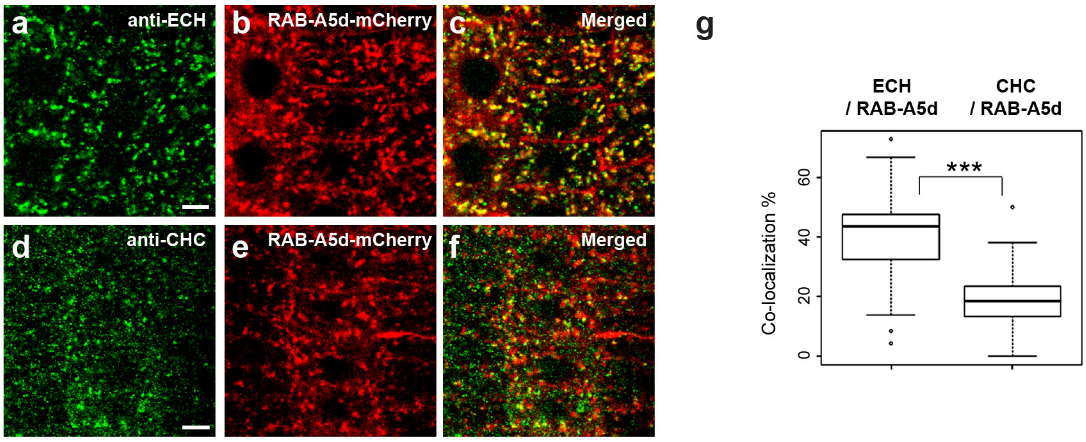

Supplementary Fig. 8. RAB-A5d localizes at SVs sites of TGN rather than CCVs sites of TGN. (**a**-**f**) Co-localization of RAB-A5d-mCherry (**b**, **e**) with either the marker of SVs sites of TGN, ECH (**a**), or the marker of CCVs sites of TGN, CHC (**d**). (**c**, **f**) Merged pictures of **a**, **b** and **d**, **e** respectively. (**g**) Quantification of co-localisation events show a strong match of RAB-A5d with TGN-associated-SVs while weaker co-localisation is detected with TGN-associated-CCVs (n = 40 cells distributed over 10 roots for each experiment, 3 biological replicates). Statistics were done by two-sided Welch two sample T-Test, *** p-value < 0.001. All scale bars are 5 µm.

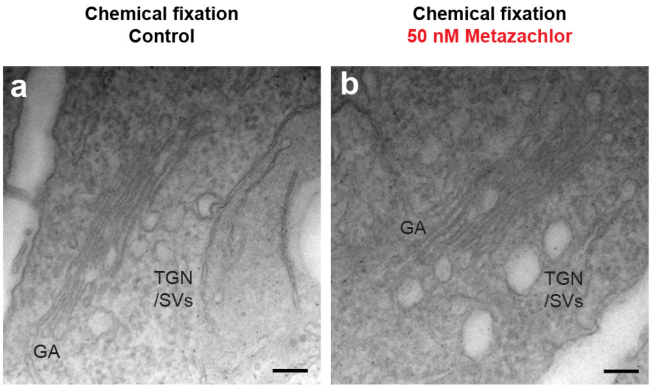

Supplementary Fig. 9. Electron microscopy of TGN membrane structure in *Arabidopsis* roots chemically fixed. (**a**) Untreated sample showing Golgi apparatus (GA) and the secretory vesicles visible as a tubulo-vesiculated membrane network (SVs/TGN) at the *trans*-side of the Golgi. (**b**) 50 nM metazachlor-treated sample showing Golgi apparatus and swollen TGN-associated secretory vesicles. Scale bars are 100 nm.
